# The San Diego 2007 wildfires and Medi-Cal emergency department presentations, inpatient hospitalizations, and outpatient visits: An observational study of smoke exposure periods and a bidirectional case-crossover analysis

**DOI:** 10.1371/journal.pmed.1002601

**Published:** 2018-07-10

**Authors:** Justine A. Hutchinson, Jason Vargo, Meredith Milet, Nancy H. F. French, Michael Billmire, Jeffrey Johnson, Sumi Hoshiko

**Affiliations:** 1 Environmental Health Investigations Branch, California Department of Public Health, Richmond, California, United States of America; 2 Climate Change and Health Equity Program, California Department of Public Health, Richmond, California, United States of America; 3 Michigan Tech Research Institute, Michigan Technological University, Ann Arbor, Michigan, United States of America; 4 Epidemiology & Immunization Services Branch, Health & Human Services Agency, County of San Diego, San Diego, California, United States of America; Africa Program, UNITED STATES

## Abstract

**Background:**

The frequency and intensity of wildfires is anticipated to increase as climate change creates longer, warmer, and drier seasons. Particulate matter (PM) from wildfire smoke has been linked to adverse respiratory and possibly cardiovascular outcomes. Children, older adults, and persons with underlying respiratory and cardiovascular conditions are thought to be particularly vulnerable. This study examines the healthcare utilization of Medi-Cal recipients during the fall 2007 San Diego wildfires, which exposed millions of persons to wildfire smoke.

**Methods and findings:**

Respiratory and cardiovascular International Classification of Diseases (ICD)-9 codes were identified from Medi-Cal fee-for-service claims for emergency department presentations, inpatient hospitalizations, and outpatient visits. For a respiratory index and a cardiovascular index of key diagnoses and individual diagnoses, we calculated rate ratios (RRs) for the study population and different age groups for 3 consecutive 5-day exposure periods (P1 [October 22–26], P2 [October 27–31], and P3 [November 1–5]) versus pre-fire comparison periods matched on day of week (5-day periods starting 3, 4, 5, 6, 8, and 9 weeks before each exposed period). We used a bidirectional symmetric case-crossover design to examine emergency department presentations with any respiratory diagnosis and asthma specifically, with exposure based on modeled wildfire-derived fine inhalable particles that are 2.5 micrometers and smaller (PM_2.5_). We used conditional logistic regression to estimate odds ratios (ORs), adjusting for temperature and relative humidity, to assess same-day and moving averages. We also evaluated the United States Environmental Protection Agency (EPA)’s Air Quality Index (AQI) with this conditional logistic regression method. We identified 21,353 inpatient hospitalizations, 25,922 emergency department presentations, and 297,698 outpatient visits between August 16 and December 15, 2007. During P1, total emergency department presentations were no different than the reference periods (1,071 versus 1,062.2; RR 1.01; 95% confidence interval [CI] 0.95–1.08), those for respiratory diagnoses increased by 34% (288 versus 215.3; RR 1.34; 95% CI 1.18–1.52), and those for asthma increased by 112% (58 versus 27.3; RR 2.12; 95% CI 1.57–2.86). Some visit types continued to be elevated in later time frames, e.g., a 72% increase in outpatient visits for acute bronchitis in P2. Among children aged 0–4, emergency department presentations for respiratory diagnoses increased by 70% in P1, and very young children (0–1) experienced a 243% increase for asthma diagnoses. Associated with a 10 μg/m^3^ increase in PM_2.5_ (72-hour moving average), we found 1.08 (95% CI 1.04–1.13) times greater odds of an emergency department presentation for asthma. The AQI level “unhealthy for sensitive groups” was associated with significantly elevated odds of an emergency department presentation for respiratory conditions the day following exposure, compared to the AQI level “good” (OR 1.73; 95% CI 1.18–2.53). Study limitations include the use of patient home address to estimate exposures and demographic differences between Medi-Cal beneficiaries and the general population.

**Conclusions:**

Respiratory diagnoses, especially asthma, were elevated during the wildfires in the vulnerable population of Medi-Cal beneficiaries. Wildfire-related healthcare utilization appeared to persist beyond the initial high-exposure period. Increased adverse health events were apparent even at mildly degraded AQI levels. Significant increases in health events, especially for respiratory conditions and among young children, are expected based on projected climate scenarios of wildfire frequency in California and globally.

## Abstract 2

Sumi Hoshiko and colleagues present data on Medi-Cal inpatient and outpatient hospitalisations following the 2007 San Diego wildfires and predict an increase in respiratory conditions as wildfires increase due to climate change.

## Author summary

**Why was this study done?:**

- Large wildfires are becoming more frequent and are expected to increase with climate change. Smoke from wildfires can cause health problems, especially for children, older persons, and people who already have respiratory or heart problems.
- Researchers had access to data on emergency department visits, hospitalizations, and outpatient visits from California’s Medicaid program, Medi-Cal. This allowed for analysis of the effects of wildfire among a particularly vulnerable population, which included a large proportion of young children. It also provided an opportunity to examine changes in outpatient visits.
- Researchers were able to look at health problems during the time when the wildfire smoke was most intense and also at later periods to see if people had health problems that may take more time to develop. They chose to study a very large wildfire that happened in San Diego County in 2007.

**What did the researchers do and find?:**

- During the peak fire period, emergency department visits for respiratory conditions increased by 34% and visits for asthma by 112%. There was no change in visits for heart-related problems.
- Some healthcare visit types remained high even after the peak fire period. For example, outpatient visits for acute bronchitis were 72% above the usual rate in the 5-day period following the peak fire period.
- Young children had bigger increases in visits during the peak fire period than older age groups. Children aged 0–4 had a 136% increase in emergency department visits for asthma, and very young children aged 0–1 experienced a 243% increase.
- Researchers studied how health visits changed on days with more intense smoke using data from smoke models. Emergency department visits for asthma went up 73% on days following an air quality day designated as “unhealthy for sensitive populations,” based on wildfire smoke and using the United States Environmental Protection Agency (EPA)'s Air Quality Index (AQI) air pollution levels as a guide.

**What do these findings mean?:**

- We expect increases in respiratory problems during wildfires, possibly even at mildly degraded levels of air quality. People may continue to seek care for some persisting conditions.
- Young children appear at highest risk for respiratory problems during a wildfire, which is cause for particular concern because of the potential for long-term harm to children’s lung development.
- The risk of future wildfires on the health of Californians will continue to be shaped by global climate change, as well as the anticipated growth of vulnerable subpopulations. Planning to protect the health of vulnerable populations is important.

## Introduction

Large forest fires have become more frequent in the Western United States since the 1980s [1–3]. Under most future climate scenarios, the frequency and size of wildfires in the southwestern states are expected to increase [4]. Climate models predict up to a 74% increase in area burned in California and a possible doubling of wildfire emissions by the end of the century [5]. Wildfires release large amounts of particulate matter (PM) and other toxic substances into the air, including carbon dioxide, carbon monoxide, and methane [6–7]. In the coterminous US, yearly emissions of fine PM from wildfire smoke are estimated to be between 118,000 and 986,000 metric tons and carbon dioxide emissions between 24 and 134 million metric tons, in addition to other compounds and gases [6]. In 2012, wildfires contributed 20% of the fine particulate emissions in the US [8].

Smoke from fires can be transported to affect populations far downwind [9]. Projected trends in climate change show that, globally, the number of people who will experience adverse health effects from wildfires is increasing [10–12]. The number of persons who are vulnerable is also expanding because more people live near wildlands [13].

Wildfire smoke exposures have been associated with adverse health outcomes, including premature death and increased inpatient hospitalizations and emergency department presentations [14–16]. Smoke from wildfires produces inhalable particles that are 10 micrometers and smaller (PM_10_) and fine inhalable particles that are 2.5 micrometers and smaller (PM_2.5_). PM_10_ and PM_2.5_ have consistently been linked to respiratory outcomes, particularly asthma exacerbations [15–17] and in some studies, cardiovascular outcomes [17–20]. Relatively few studies of wildfire smoke have examined the health effects on vulnerable populations. However, the nature and intensity of health impacts are expected to depend on characteristics of the receptor population [16,17,21]. Research on vulnerability to ambient air pollution has identified subpopulations with increased susceptibility to the effects of PM; these include persons with chronic diseases [22], as well as older adults, children, and possibly those with lower education, income, and employment status [23]. Although PM of wildfire origin differs from ambient air pollution in composition and exposure patterns, current research suggests that elderly and young populations will also be especially vulnerable to wildfire-derived PM [16,17,24]. Children warrant particular concern because their lungs are still developing, and exposure to ambient air pollution has been shown to permanently impair lung function [25].

Individual socioeconomic position or status (SES) factors such as personal income and education are accompanied by a broad range of factors that influence health, including prevalent comorbid conditions such as respiratory and cardiovascular diseases, as well as access to healthcare, social stress, and environmental quality of the community [26]. Often, these factors are difficult to isolate.

California’s Medicaid program, Medi-Cal, is a public health insurance program covering health services for low-income individuals, including seniors, persons with disabilities, families with children, children in foster care, pregnant women, and childless adults with incomes below 138% of the federal poverty level. These eligibility criteria create a population that tends to be focused on low-income women and children, plus others with varying disabilities. Beginning at age 65, Medicare is available regardless of income, so for this group, Medi-Cal only pays secondarily or for certain services not covered by Medicare.

In this study, we investigated change in healthcare utilization—including differential health responses by age groups and type of health service—related to wildfire smoke exposure from a large complex of fires in San Diego County in 2007 within a vulnerable population, Medi-Cal beneficiaries who resided in San Diego County at the time.

## Methods

### Study area and design

In late October of 2007, a complex of fires burned nearly 1 million acres in San Diego county, resulting in the evacuation of an estimated 515,000 county residents and numerous road, school, and business closures [27]. San Diego county had a population of 3,095,342 according to the 2010 US Census [28], with the population concentrated along the coastal areas.

Medi-Cal beneficiaries numbered 345,257 in San Diego County in July 2007 [29]. Medi-Cal administrative claims data were obtained from the California Department of Health Care Services’ (DHCS) Management Information System/Decision Support System (MIS/DSS) data warehouse for San Diego County for the period of August 1 through December 31, 2007 to accommodate reference dates surrounding the late-October fire period.

We conducted 2 types of analyses. The first was a county-wide analysis of Medi-Cal claims data, which compared rates for emergency department presentations, inpatient hospitalizations, and outpatient visits during the fires with reference periods. The second was a case-crossover analysis that examined exposures by residential zip code and emergency department presentations with respiratory diagnoses.

For the county-wide analysis, we identified October 22–26 as the peak fire-exposure period (P1) based on a previous study that analyzed this fire using data from the BioSense Platform, an integrated national syndromic surveillance system [30]. We defined 2 following periods, P2 (October 27–31) and P3 (November 1–5), for analysis in order to identify any health outcomes that might be sensitive to cumulative or lagged exposure to wildfire smoke.

For the case-crossover analyses of exposure to varying concentrations of PM_2.5_, the population was limited to those beneficiaries with a valid San Diego County zip code listed for their residential address. Where possible, post office-box–only zip codes were mapped to real-address zip codes in the same subregion, municipality, and neighborhood. Exposures were based on the modeled PM_2.5_ for these 101 real-address zip codes.

### Environmental data

Wildfire PM_2.5_ concentrations were estimated through the use of coupled models of wildfire smoke emissions and atmospheric dispersion [31]. Spatially and temporally resolved estimates of wildland fire emissions were computed using the geospatial tool Wildland Fire Emissions Information System (WFEIS); model outputs were then introduced into the meteorological atmospheric transport model Hybrid Single-Particle Lagrangian Integrated Trajectories (HYSPLIT) to produce PM concentration estimates computed to a 0.01-degree grid (approximately 1 km^2^) on an hourly basis. Hourly model outputs were used to estimate daily average wildfire PM_2.5_ concentrations (μg/m^3^) by zip code, as described previously [31]. All analyses in this study are based on PM originating from wildfire sources, so all PM in this manuscript refers to wildfire-only PM. We interpolated relative humidity and temperature data from a Remote Automated Weather Station database to county subregional areas for the period of August to November 2007 (environmental data availability period).

### Health data

Medi-Cal dataset variables included county of residence and home zip code of the patient, date of the medical visit, general type of service provided, where the visit occurred, classification of the provider (i.e., hospital, emergency department, outpatient, excluding claims related to nursing homes, etc.), and diagnosis that was being treated (by International Classification of Diseases [ICD]-9 code, up to 2 diagnoses per claim). Patient demographic variables included sex and age. A unique, de-identified beneficiary code (beneficiary ID) was provided with the dataset; names were not included. Eligible subjects were San Diego County residents who had a qualifying Medi-Cal fee-for-service claim during the study period. Qualifying claims included those for inpatient hospitalizations, emergency department presentations, and outpatient visits (clinic and physician office visits). The DHCS Data and Research Committee and California’s Health and Human Services Agency’s Committee for the Protection of Human Subjects approved the study protocol. We performed data management and analysis using SAS version 9.4 (SAS Institute; https://www.sas.com/en_us/home.html) and Excel for Mac version 14.4.3 (Microsoft, https://www.microsoft.com/en-us/).

### Identification and description of beneficiaries

The beneficiary ID linked all claims records for each beneficiary. Beneficiaries aged 65 and above were excluded from the study because claims for these beneficiaries were not adequately represented in the Medi-Cal data due to their dual eligibility for Medicare and Medi-Cal.

### Identification of episodes of care

Episodes of care (“encounters”) were identified from the subset of records with at least one valid diagnosis code. For each beneficiary, inpatient status was assessed for each day from August 1 through December 31, 2007. Inpatient hospitalizations were identified as periods of one or more contiguous days with associated inpatient claims records; the start date of the earliest record was used as the admission date. Emergency department claims records for each beneficiary from the same date were grouped together into a single episode of care. Overnight emergency department presentations were identified, and records from both those dates were grouped into a single episode of care. Physician office and clinic claims records for each beneficiary from the same date were grouped together into a single episode of care, referred to hereafter as outpatient visits. To reduce misclassification of inpatient diagnosis, errors in ascertainment of inpatient status, and errors in date of inpatient admission, the episodes-of-care dataset was limited to episodes with admission during the period of August 16 to December 15, 2007 (encounter data availability period).

Episodes of care were identified as being related to the outcomes of interest based on the primary and secondary diagnoses from any associated claims records, except inpatient hospitalizations, which were limited to claims records from the first 14 days of the hospitalization. Encounters for components of a respiratory index and a cardiovascular index were identified as outcomes for analysis, based on ICD-9 coding in a previous study of a large wildfire event in California (Table 1) [32]. The respiratory index included asthma, acute bronchitis, chronic obstructive pulmonary disease (COPD), bronchitis—not otherwise specified, pneumonia, upper respiratory infections, cystic fibrosis, bronchiectasis, extrinsic allergic alveolitis, respiratory symptoms, and other acute and subacute respiratory conditions caused by exposure to fumes, vapors, or external agents. The cardiovascular index included ischemic heart disease, dysrhythmia, congestive heart failure, cerebrovascular disease including stroke, and peripheral vascular disease. We also examined total visits (all-cause) for each healthcare setting to provide context for results for the outcomes of interest.

**Table 1:** ICD-9 codes used to classify respiratory and cardiovascular disorders.

Condition	ICD-9 codes
***Respiratory index (all respiratory codes below)***	
Asthma	493
Acute bronchitis	466
COPD	491, 492, 496
Bronchitis—not otherwise specified	490
Pneumonia	480–487
Upper respiratory infections	460–464
Cystic fibrosis	277
Bronchiectasis	494
Extrinsic allergic alveolitis	495
Respiratory symptoms	786
Other acute and subacute respiratory conditions caused by exposure to fumes, vapors or external agents	506, 508
***Cardiovascular index (all cardiovascular codes below)***	
Ischemic heart disease	410–414
Dysrhythmia	426, 427
Congestive heart failure	402–428
Cerebrovascular disease including stroke	430–438
Peripheral vascular disease	450–459

Abbreviations: COPD, chronic obstructive pulmonary disease; ICD, International Classification of Diseases.

### Data analysis

#### County-wide results by exposure periods

For the entire study population (ages 0–64), rate ratios (RRs) were calculated by counting occurrences of the outcomes of interest during the 5-day county-wide exposed periods P1 (October 22–26; highest exposures), P2 (October 27–31; lower exposures and lagged or cumulative effects), and P3 (November 1–5; lagged effects and cumulative effects) and comparing them with occurrences of the same outcome during six 5-day comparison periods, matched on day of week (5-day periods starting 3, 4, 5, 6, 8, and 9 weeks before each exposed period; weeks 1 and 2 were excluded because, for P2 and P3, they would have overlapped with P1, and week 7 was excluded to prevent comparing P1 to the Labor Day holiday). For 5 age groups of interest (0–1 years, 2–4 years, 0–4 years, 5–17 years, and 18–64 years), RRs were calculated by counting occurrences of the outcomes of interest during P1 and comparing them with occurrences of the same outcome during eight 5-day comparison periods, matched on day of week (5-day periods starting 1, 2, 3, 4, 5, 6, 8, and 9 weeks before the exposed period). We calculated Mid-P 95% confidence intervals (CIs) for RRs based on fewer than 10 events (exposed or unexposed) using OpenEpi version 3.01. For RRs based on 10 or more events (exposed and unexposed), we estimated 95% CIs using large-sample statistics for person-time RRs [33], with the following formula:

$$95\%\;\text{CI}={\text{e}}^{\left[\text{ln}\;(\text{RR})\pm 1.96*\surd (1/{\text{A}}_{1}+1/{\text{A}}_{0})\right]},$$

where A_1_ is the number of events in the exposed period and A_0_ is the number of events in the control period.

Statistical significance of increases and decreases in rates were determined from the 95% confidence limits of the RR testing exclusion of 1 (prior to rounding). Changes in the incidence of an outcome in the fire period relative to the control period were calculated using the following formula: (RR − 1) × 100%.

#### Case-crossover analysis of acute respiratory events related to PM_2.5_ concentrations

The bidirectional symmetric case-crossover method [34], a statistical technique suited to examine acute effects of air pollution and effect modification for variables at the individual level, was used for this analysis. The case-crossover study is a matched design in which each case subject (on a different day or days) serves as its own control, thereby adjusting for time-invariant confounders, both known and unknown. The bidirectional symmetric design selects 2 control days from equal distances before and after the event, providing adequate control for both long-term trends and seasonality. Seasonality is of particular concern when examining respiratory health outcomes. To adjust for potential confounding by day of the week, we selected control days on the same day of the week as the case. Based on our analysis of emergency department presentations for respiratory diagnoses and asthma in P3, we expected negligible elevation in these outcomes 10–15 days after exposures. Therefore, we eliminated correlation in the exposure of interest between case days and control days by setting the interval between case days and control days to 14 days, selecting control days 2 weeks before and 2 weeks after cases. Based on the availability of episode-of-care and environmental data, the need to model lagged exposures, and the need to use exposure data from 14 days before and after each event modeled, events spanned the period from September 15 to November 15, and control days spanned the period from September 1 to November 29.

After creating a case-crossover matrix with as many strata as events, we used the SAS procedure LOGISTIC to conduct conditional logistic regressions of 2 outcomes separately—emergency department presentations for respiratory diagnoses and for asthma. PM_2.5_ was scaled to represent increased odds of the inpatient hospitalization per 10 μg/m^3^ increase. Using SAS, we obtained risk estimates in the form of an odds ratio (OR), corresponding 95% CI and Wald *p*-values, and Akaike information criteria (AIC) values for each model.

To determine the best model fit based on the AIC, several exposures were considered, including single-day averages of the same day (24 hour), averages of the same day and the previous day (48-hour), and averages of the same day and 2 previous days (72-hour), all adjusted for temperature and humidity. We evaluated possible effect modification by age by adding an interaction term of PM by age category and assessing significance. We also stratified by sex and re-examined significance of the age interaction.

To investigate the usefulness of existing public health recommendations, we categorized 24-hour average PM_2.5_ concentrations according to the categories of the Air Quality Index (AQI), an index created by the US Environmental Protection Agency (EPA) for communicating daily air quality risks to the public [35]. The AQI values are ranked into categories—good, moderate, unhealthy for sensitive groups, unhealthy, very unhealthy, and hazardous—each with corresponding recommendations for protecting health [36]. For the outcomes of the respiratory index emergency department presentations, we performed conditional logistic regression, adjusting for temperature and relative humidity and calculating ORs relative to the reference category of “good.”

In our original data analysis plan (no changes made to the IRB submission, S1 Protocol), we had proposed several additional analyses that were not ultimately conducted. Because we had anticipated finding overall excesses, we had planned to statistically screen multiple diagnosis codes and groupings in order to determine which outcomes were driving the elevations. Based on finding that the excess visits were concentrated among the respiratory diagnoses that we were already evaluating separately, no additional screening was warranted. We also had proposed calculating the cost burden but, due to time considerations, decided not to pursue these additional analyses.

## Results

### Population

During the health data availability period of August 1 to December 31, 2007, there were a total of 5,454,360 Medi-Cal claims for San Diego beneficiaries, derived from 217,067 residents with at least one claim of any type (not limited to the claim types we examined). We excluded 40,216 residents aged 65 and above. After these exclusions, during the fire period of October 22–26, 2007, there were 26,556 San Diego County residents with at least one Medi-Cal claim (15.0% of beneficiaries). The individuals with at least one claim during the health data availability period and fire period are described by age, sex, and race/ethnicity (Table 2).

**Table 2:** Age, sex, and race/ethnicity of Medi-Cal beneficiaries under age 65 with fee-for-service claims during health data availability period (August 1–December 31, 2007) and peak fire period (October 22–26, 2007) in San Diego County.

	Data Availability Period (Aug 1–Dec 31, 2007)		Fire Period (Oct 22–26, 2007)	
	*N*	%	*N*	%
**Total**	176,851	100	26,556	100
**Age**				
0–1	24,490	13.8	2,191	8.3
2–4	15,546	8.8	1,197	4.5
5–17	42,548	24.1	4,004	15.1
18–64	94,259	53.3	19,162	72.2
Unknown/missing	8	0.00	2	0.00
**Sex**				
Female	110,178	62.3	16,099	60.6
Male	66,317	37.5	10,427	39.3
Unknown/missing	356	0.2	30	0.1
**Race/ethnicity**				
Asian/Pacific Islander	8,969	5.1	1	6.7
Black	13,807	7.8	2,575	9.7
Hispanic	77,447	43.8	9,984	37.6
Native American	821	0.5	136	0.5
White	36,306	20.5	8,014	30.2
Other/unknown	39,501	22.3	4,056	15.3

### Episodes of care

Among our study population and during the period of August 16 to December 15, 2007, we identified 25,000 emergency department presentations, 17,009 inpatient hospitalizations, and 269,842 outpatient visits. Young children aged 0–4 comprised 14.4% of inpatient hospitalizations, 15.1% of emergency department presentations, and 28.8% of outpatient visits. Very young children (aged 0–1) accounted for 12.8% of inpatient hospitalizations, 10.8% of emergency department presentations, and 15.8% of outpatient visits.

### Exposures

Wildfire-derived PM_2.5_ concentrations are shown in Table 3. During the most intense initial period of the firestorm P1, the mean of the 24-hour average PM_2.5_ concentrations of all the zip codes was 89.1 μg/m^3^. The highest of all the zip codes’ daily averages occurred during this window of time, 803.1 μg/m^3^. In comparison, the US EPA 24-hour air quality standard for PM_2.5_ is 35 μg/m^3^, and concentrations over 250 μg/m^3^ correspond to AQI level “hazardous.”

**Table 3:** Summary of modeled wildfire emissions: mean 24-hour, maximum 24-hour, and percentiles of 24-hour wildfire PM_2.5_ concentrations across zip codes and dates during study periods in San Diego County in 2007.

	24-Hour Average PM_2.5_ (μg/m^3^) for Zip Codes by Exposure Period		
Measure	P1 (Day 1–5)	P2 (Day 6–10)	P3 (Day 11–15)
**Daily mean**	89.1	9.33	0.26
**Daily maximum**	803.1	283.9	5.72
**Percentile**			
5	0.2	0.0	0.0
25	7.0	0.0	0.0
50	39.9	0.68	0.16
75	131.5	13.17	0.3
95	333.1	40.5	1.05

Abbreviation: PM_2.5_, fine inhalable particles that are 2.5 micrometers and smaller.

**Fig 1:**
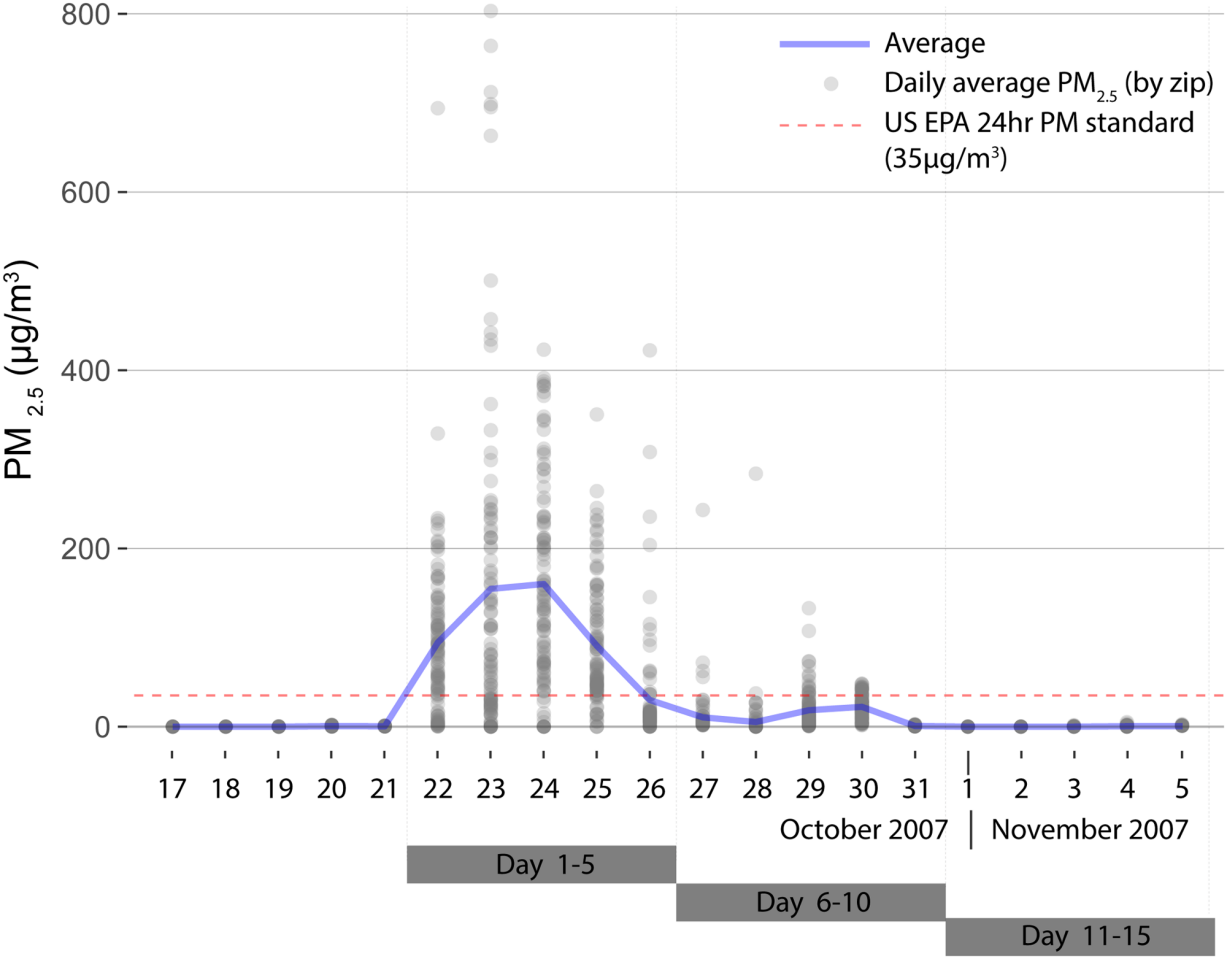
Wildfire PM_2.5_ by day in San Diego County zip codes during 2007 wildfires. Daily average wildfire PM_2.5_ for each of 101 zip codes in San Diego County for a period just prior to the 2007 Firestorm and for the 5-day windows of time following the start of the fires on October 22. The average for all zip codes is shown in blue, and the US EPA 24-hour PM_2.5_ standard is in red. PM, particulate matter; PM_2.5_, fine inhalable particles that are 2.5 micrometers and smaller; US EPA, Environmental Protection Agency.

**Fig 2:**
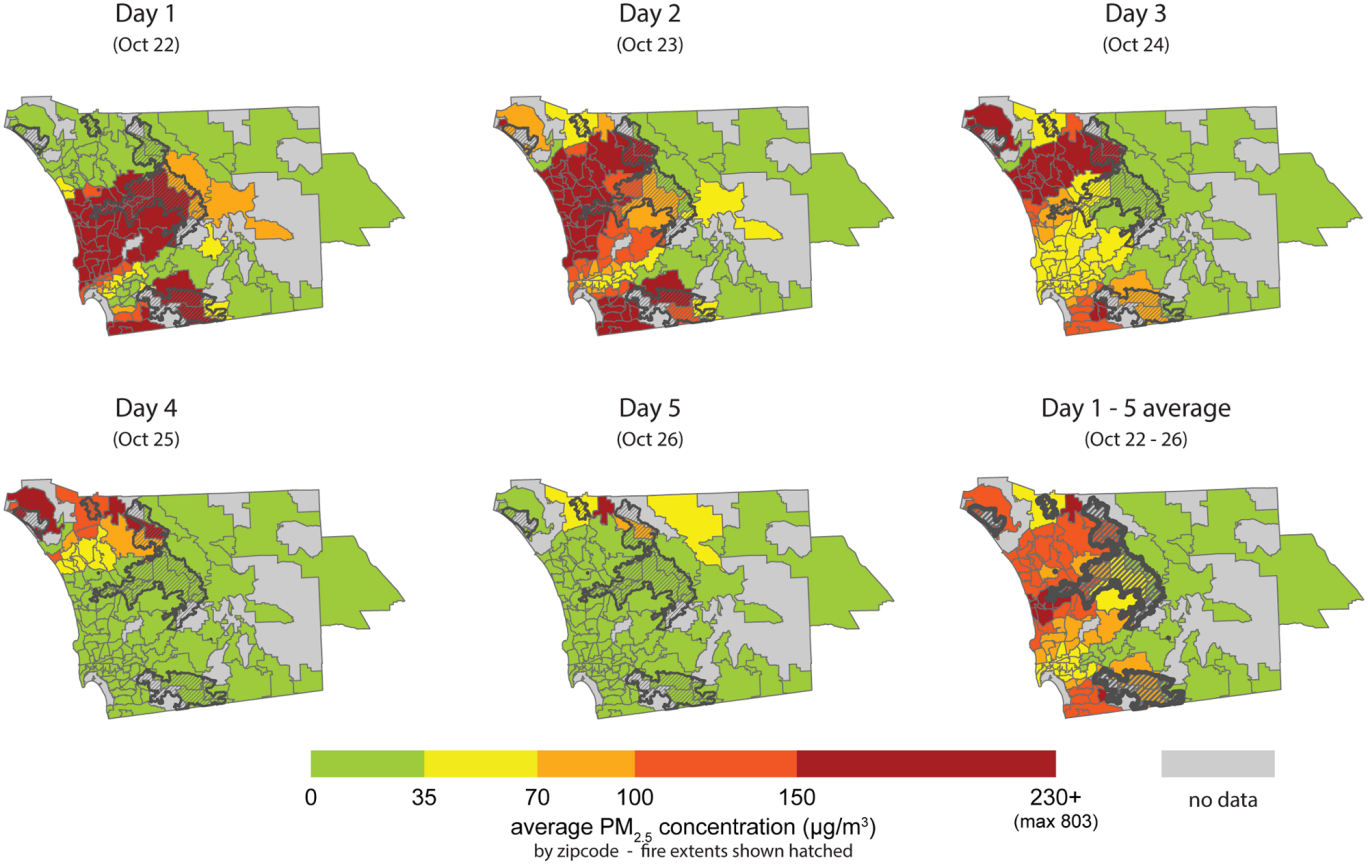
Map of San Diego County wildfire PM_2.5_ by zip code, October 22–26, 2007 fire period. Maps show zip code mean of average daily PM_2.5_ values across the 5-day fire-exposure period. Green indicates satisfactory levels according to the US EPA’s 24-hour standard. Fire extent is hatched. PM_2.5_, fine inhalable particles that are 2.5 micrometers and smaller; US EPA, US Environmental Protection Agency.

Estimated average daily wildfire PM_2.5_ concentrations by zip code through the course of the fire period are shown in Fig 1. Concentrations spiked sharply on October 22 and continued through the initial 5-day fire period, then declined. The mean PM_2.5_ concentration on the first day of the 5-day fire period was 160 μg/m^3^ (AQI “very unhealthy”), which then dropped to 29.9 μg/m^3^ on the 5th day (AQI “moderate”). The fire boundaries and daily average PM_2.5_ concentrations by zip code in San Diego County are mapped for the 5-day exposure period (P1) (Fig 2).

### County-wide results by exposure period

#### Total visits

**Fig 3:**
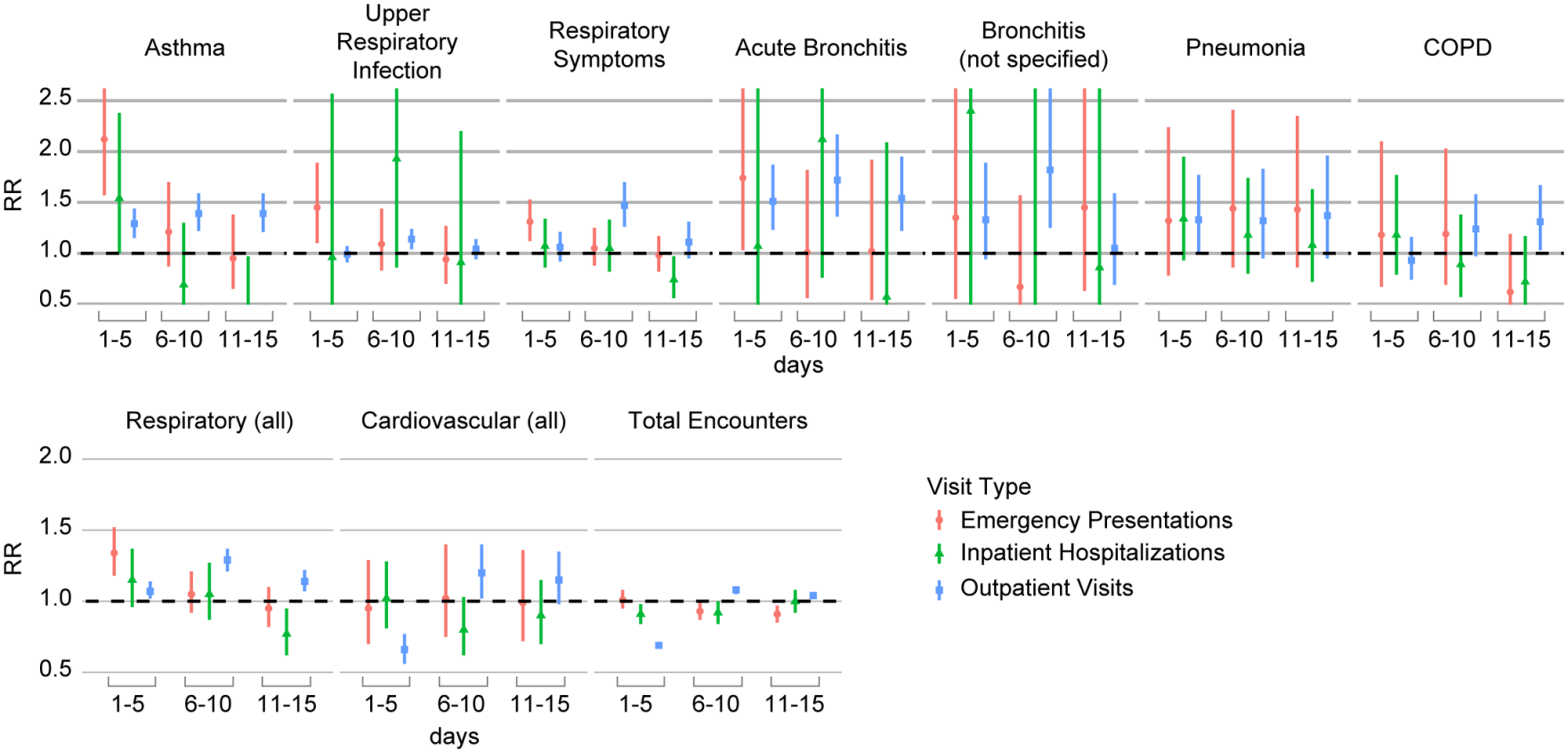
Respiratory and cardiovascular healthcare encounters in San Diego County during 2007 fire period. RRs for the 5-day periods starting from October 22 and for claims related to emergency department presentations (red, circle), inpatient hospitalizations (green, triangle), and outpatient visits (blue, square). The top row shows encounters for specific respiratory diagnoses. The bottom row shows encounters for the respiratory index, cardiovascular index, and total encounters (all diagnoses). COPD, chronic obstructive pulmonary disease; RR, rate ratio.

During P1 (October 22–26), there were 1,071 emergency department presentations, 725 inpatient hospitalizations, and 10,822 outpatient visits. RRs for the 5-day periods P1–P3 for total (all-cause) encounters and encounters for selected respiratory and cardiovascular diagnoses are shown in Fig 3 (S1 Table). Relative to the 6 reference periods, total emergency department presentations did not change significantly during P1 (1,071 versus 1,062.2; RR 1.01; 95% CI 0.95–1.08); inpatient hospitalizations (725 versus 797.8; RR 0.91; 95% CI 0.84–0.98) and outpatient visits (10,822 versus 15,790.7; RR 0.69; 95% CI 0.67–0.70) decreased significantly.

#### Respiratory outcomes

Despite the overall pattern of no change or deficits in total healthcare encounters, the index of respiratory diagnoses increased across all healthcare settings, with the largest magnitude observed in emergency department presentations (e.g., P1: 288 versus 215.3; RR 1.34; 95% CI 1.18–1.52).

Of the outcomes we studied, the elevations in asthma encounters were the most pronounced. In P1, excess asthma encounters were evident across all healthcare settings, although the relationship was strongest in emergency department presentations (58 versus 27.3; RR 2.12; 95% CI 1.57–2.86).

Infectious respiratory outcomes—upper respiratory infections, bronchitis, and pneumonia—increased in some healthcare settings during P1. Emergency department presentations for upper respiratory infections increased (RR 1.45; 95% CI 1.10–1.89), but not outpatient visits (RR 0.99; 95% CI 0.91–1.07). Outpatient visits for acute bronchitis were also significantly elevated in P1 (RR 1.51; 95% CI 1.23–1.87). Inpatient hospitalizations for COPD increased nonsignificantly in P1 (RR 1.18; 95% CI 0.79–1.77).

In general, similar types of health conditions were elevated in P2 and P3 as in P1. However, although observed increases in emergency department presentations and inpatient hospitalizations generally decreased after P1, elevations for some respiratory outcomes persisted beyond this initial high-exposure period. For example, although based on small numbers (<50), RRs for pneumonia were elevated in P1–P3 across all settings. However, some outpatient visits increased in the later time frames. Outpatient visit increases for the respiratory index appeared larger in P2 (RR 1.29; 95% CI 1.21–1.37) and P3 (RR 1.14; 95% CI 1.07–1.22) than in P1 (RR 1.07; 95% CI 1.02–1.14). Outpatient visits for individual respiratory diagnoses showed excess visits in P2, which were generally higher than in P1. For example, outpatient visits for acute bronchitis were elevated in P2 (RR 1.72; 95% CI 1.36–2.17). For COPD, we found emergency department presentations to be elevated in P1 (RR 1.18; 95% CI 0.67–2.10) and P2 (RR 1.19; 95% CI 0.69–2.03), although without reaching statistical significance. A reverse pattern was seen for COPD outpatient visits, for which an initial nonsignificant deficit in P1 and P2 turned to a significant excess in P3 (RR 1.31; 95% CI 1.03–1.67), although this could also be due, at least in part, to people making up earlier missed appointments.

#### Cardiovascular index

RRs for the cardiovascular index tended towards null, although an increase was observed in outpatient visits in P2. Although the numbers of encounters with cardiovascular diagnoses were small, the pattern of the cardiovascular index appeared similar to that of total visits. Although based on very small numbers (<20), the few cardiovascular conditions with RR >1 in the context of emergency department presentations and inpatient hospitalizations included dysrhythmia and stroke.

#### Young children

**Fig 4:**
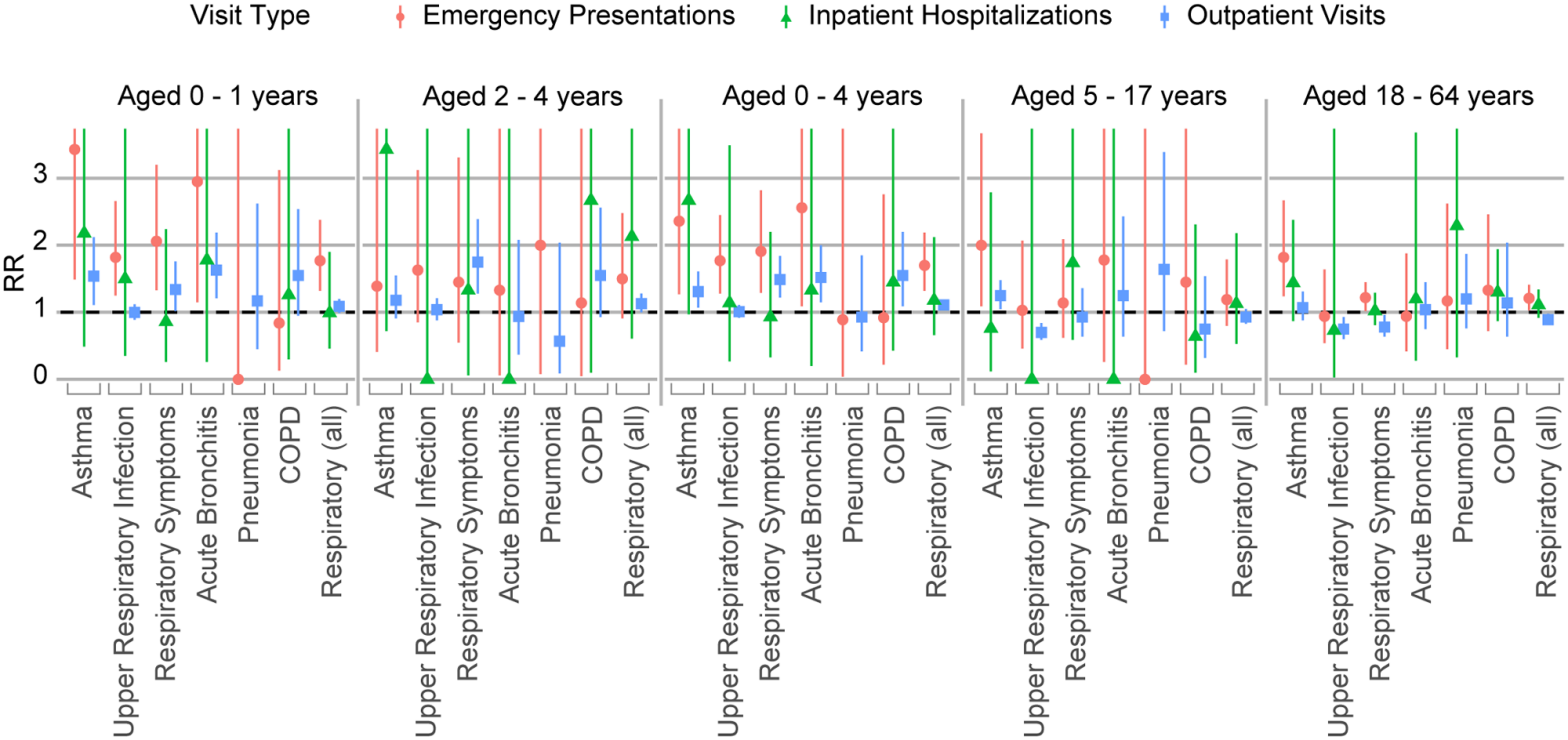
Respiratory healthcare encounters, age-specific results in San Diego County during 2007 fire period. RRs by age group (young children aged 0–1, 2–4, 0–4; older children aged 5–17; and adults under age 65) for the 5-day exposure period starting from October 22 for emergency department presentations, inpatient hospitalizations, and outpatient visits. COPD, chronic obstructive pulmonary disease; RR, rate ratio.

Relative risks by age group highlight the vulnerable status of young children (Fig 4; S2 Table). In P1, young children aged 0–4 showed significantly elevated emergency department presentations for respiratory diagnoses (RR 1.70; 95% CI 1.32–2.19), asthma (RR 2.36; 95% CI 1.27–4.39), upper respiratory infections (RR 1.77; 95% CI 1.28–2.45), and respiratory symptoms (RR 1.91; 95% CI 1.29–2.82). Although based on small numbers (<10), emergency department presentations for acute bronchitis (RR 2.56; 95% CI 1.09–5.54) were elevated in P1 for these young children.

Among children aged 0–4, although there was a deficit in total outpatient visits in P1, outpatient visits for the respiratory index (RR 1.11; 95% CI 1.03–1.19), respiratory symptoms (RR 1.49; 95% CI 1.22–1.84), and acute bronchitis (RR 1.52; 95% CI 1.15–2.00) were significantly elevated. Outpatient visits for pneumonia were also elevated (RR 1.55; 95% CI 1.09–2.20). Although based on very small numbers (<20) and not statistically significant, inpatient hospitalizations for respiratory diagnoses (RR 1.18; 95% CI 0.66–2.12) and for asthma (RR 2.67; 95% CI 0.97–6.53) among children aged 0–4 were elevated in P1.

RRs for children under age 2 (aged 0–1) appeared generally higher than those for young children aged 2–4. The increase in emergency department presentations with respiratory diagnoses appeared greater among children aged 0–1 (RR 1.77; 95% CI 1.32–2.38) than 2–4 (RR 1.50; 95% CI 0.91–2.48). Although based on very small numbers (<10), emergency department presentations for asthma (RR 3.43; 95% CI 1.49–7.38) and acute bronchitis (RR 2.95; 95% CI 1.15–6.85) were elevated among children aged 0–1.

#### Older children and adults

Unlike younger children, children aged 5–17 in P1 had significantly fewer total encounters across emergency department, inpatient hospital, and outpatient settings versus reference periods. However, for asthma, children aged 5–17 had increased rates of outpatient visits in P1 (RR 1.25; 95% CI 1.05–1.48). Among adults aged 18–64, emergency department presentations for respiratory diagnoses (RR 1.21; 95% CI 1.03–1.41), asthma (RR 1.82; 95% CI 1.24–2.67), and respiratory symptoms (RR 1.22; 95% CI 1.02–1.45) were elevated in P1.

### Conditional logistic regression of emergency department presentations for respiratory diagnoses and asthma

In multivariate models adjusted for daily temperature and relative humidity, an increase in the average PM_2.5_ of 10 μg/m^3^ for the daily, 48-hour moving, and 72-hour moving averages was associated with a 3%, 5%, and 8% increase, respectively, in the likelihood for asthma emergency department presentations, with similar but attenuated increases for respiratory visits (Table 4). ORs were greater when examining moving averages over several days, suggesting that the models were capturing cumulative and lagged effects. Square terms did not reach significance in any of the models, so linear models were selected. We did not find effect modification by age, including after stratifying by sex.

**Table 4:** Conditional logistic regression of emergency department presentations for respiratory diagnoses and asthma with wildfire PM_2.5_, and ORs adjusted for daily temperature and relative humidity in San Diego County during 2007 wildfires.

	Respiratory Index			Asthma		
PM_2.5_ Measure (10 μg/m^3^)	OR	95% Wald CL	Wald *p*-value (MLE)	OR	95% Wald CL	Wald *p*-value (MLE)
Daily average	1.02	1.01–1.04	<0.01	1.03	1.00–1.06	0.03
48-hour moving average	1.03	1.01–1.05	<0.01	1.05	1.02–1.08	<0.01
72-hour moving average	1.04	1.02–1.05	<0.01	1.08	1.04–1.13	<0.01

Abbreviations: CL, confidence limit; MLE, maximum likelihood estimate; OR, odds ratio; PM_2.5_, fine inhalable particles that are 2.5 micrometers and smaller.

### AQI: Respiratory events

Unhealthy AQI levels were associated with increased respiratory conditions in emergency department presentations, adjusting for temperature and relative humidity (Table 5). The AQI models fit best with a 1-day lag compared to same-day– or 2-day–lagged models. The AQI levels “unhealthy for sensitive groups” (OR 1.73; 95% CI 1.18–2.53) and “unhealthy” (OR 1.79; 95% CI 1.30–2.23) both were associated with significantly elevated odds of an emergency presentation the day after exposure versus the AQI level “good.” The strongest effect was seen in the same-day model for the highest exposure category, hazardous (OR 2.41; 95% CI 1.39–4.18).

**Table 5:** AQI categories—ORs from conditional logistic regression of respiratory emergency department presentations in San Diego County during 2007 wildfires.

AQI categories PM_2.5_ (μg/m^3^)	OR (95% CI) Same day	OR (95% CI) 1-day lag	OR (95% CI) 2-day lag
Good (0–12)	Reference	Reference	Reference
Moderate (12.1–35.4)	1.20 (0.91–1.59)	1.11 (0.84–1.47)	0.80 (0.59–1.08)
Unhealthy for sensitive groups (35.5–55.4)	1.43 (0.96–2.13)	1.73 (1.18–2.53)*	1.51 (1.00–2.28)*
Unhealthy (55.5–150.4)	1.27 (0.97–1.67)	1.79 (1.30–2.23)*	1.50 (1.13–1.98)*
Very unhealthy (150.5–250.4)	1.68 (1.00–2.83)	1.58 (0.93–2.68)	1.87 (1.07–3.27)*
Hazardous (≥250.5)	2.41 (1.39–4.18)*	1.28 (0.70–2.36)	1.74 (1.00–3.03)*
**Temperature**	1.00 (0.99–1.01)	1.00 (0.99–1.01)	1.00 (0.99–1.00)
**Relative humidity**	1.01 (1.00–1.01)*	1.01 (1.00–1.01)*	1.01 (1.00–1.01)*
**AIC**	5,233.2	5,228.9	5,231.8

*Statistically significant (alpha = 0.05).

Abbreviations: AIC, Akaike information criteria; AQI, Air Quality Index; CI, confidence interval; OR, odds ratio; PM_2.5_, fine inhalable particles that are 2.5 micrometers and smaller.

## Discussion

By examining multiple respiratory and cardiovascular endpoints across 3 healthcare settings and 3 exposure periods as well as for different age groups, we have compiled a relatively comprehensive view of health events during this significant wildfire complex. While outcomes such as respiratory conditions were clearly elevated, visits for other outcomes were decreased. These observed results must be viewed in the context of the extensive nature of the fire and the resulting evacuations and other disruptions. These unusual conditions likely altered healthcare-seeking behavior; residents may not have accessed healthcare other than for the most urgent conditions. A review of the relationship between the 2007 wildfires and the emergency department of the University of California, San Diego hospital found a 5.8% decrease in admissions during the fires, although the rate of patients with a chief complaint of shortness of breath increased significantly and the rate of patients who left without being seen nearly doubled [37]. Also, an assessment of the 2003 fires in San Diego noted that emergency department presentations initially declined during the fire period, corresponding to days when authorities recommended that students and employees stay home [38].

Our study examined Medi-Cal beneficiaries, a group representing a vulnerable, although fairly substantial, subset of the general population. We would anticipate their response to the health stressor of wildfire smoke to be similar in nature to the general public but possibly increased in magnitude. Asthma, as in other wildfire studies, appeared to be the most sensitive to wildfire smoke exposure [16]. Our findings support a wildfire smoke association with the infectious respiratory outcomes pneumonia, bronchitis, and upper respiratory infections despite inconsistent results from previous studies [16,39]. Airway injury from wildfire smoke exposure could predispose bacterial pneumonia. Previous wildfire studies generally have found positive associations with COPD [16]. Because COPD is a condition more prevalent in the older population, who were excluded from our analysis, this may have limited our ability to study this condition.

Similar to COPD, cardiovascular outcomes are generally more prevalent in older adults, so the absence of this population from our study is relevant here as well. However, our study is not unusual in its null cardiovascular findings for wildfire smoke exposures, despite the scientific relationship between general particulate air pollution and cardiovascular disease [40]. The reasons for this are unclear. The lower prevalence of cardiovascular events in general in comparison with respiratory conditions—along with the possibility that cardiovascular impacts from wildfire smoke may occur at a smaller magnitude than respiratory impacts—may require a larger study to detect an excess. Another factor may be that only certain diagnoses are elevated, and broadly combining all cardiovascular conditions may obscure an association. Moreover, persons with underlying cardiovascular disease may be seen for respiratory rather than cardiovascular conditions (competing diagnoses) during wildfires. Too few studies have examined specific cardiovascular outcomes to have a clear picture of which are related to wildfire exposure [15], although a recent analysis of an extensive California wildfire season provided strong evidence for increased cardiovascular risk [20].

Using sequential exposure periods during and after the peak smoke exposure allowed examination of changes over longer time frames. Studies typically do not detect any increases beyond 3 to 5 lag days. This design allowed us to show some conditions persisting over longer periods of time. Cumulative exposure may be relevant for conditions such as asthma, bronchitis, or pneumonia, which may gradually develop or worsen over time. Inhaled PM may prompt inflammation and alter immune functions, increasing susceptibility to respiratory infections. Also, patients may not seek care until their symptoms become severe.

Our examination of outpatient visits was an exception to the majority of wildfire research studies in the US, which have largely relied on inpatient hospitalization and emergency department data [15]. We noted that patients continued to seek care in outpatient settings while the initial surge in emergency department presentations was declining.

The AQI is a widely used public health tool, yet few wildfire studies have made associations with the AQI categories. The sensitivity of our study population was revealed in its response to even modestly increased concentrations of PM, as excess adverse health events began to occur at an AQI level designed to represent the first threshold at which susceptible persons are advised to consider limiting their exposure. These results provide evidence for the value of the AQI as a communication tool in conveying health risks of wildfire smoke to the public, especially because the AQI addresses the immediate day, and health events were shown to generally rise with increasing same-day AQI exposure categories.

While children are thought to be more vulnerable to effects of wildfire smoke, the literature has not been conclusive [16]. The mixed results for children may be due to different effects between very young children and older children because null results are often seen in studies that combine all ages or do not include very young children. Wildfire smoke effects among children aged 6 to 18 have been noted in a cohort study of schoolchildren who experienced increased respiratory symptoms [41]. Children’s heightened susceptibility to wildfire smoke may be related to their smaller airway size [42]. In our study, this vulnerability was most evident among the very youngest children, aged 0–1, for whom the increase in emergency department presentations during the initial wildfire period (243% increase in asthma) was the highest of any group we evaluated.

Several studies that have stratified on very young children have shown significant associations between increased respiratory admissions and/or visits and wildfire smoke exposures [32,43,44]. However, the magnitude of the association in our Medi-Cal population appears to be greater than what has been found previously in general populations, although results are not directly comparable because methods differ between studies. A study examining 0- to 4-year-olds found a potential 5% increase in the odds of physician visits for asthma, for a 60 μg/m^3^ increase in PM_10_ [41]. Our findings of 236%, 267%, and 131% increases in asthma emergency department presentations, inpatient hospitalizations, and outpatient visits, respectively, suggest a particularly high association among young children (0–4 years). This may be related to underlying vulnerability of the Medi-Cal population. Many factors may contribute to vulnerability, e.g., one study identified increased asthma risks only among children with asthma and obesity [45]. Overall, the very young in our study experienced significantly elevated risks of unusually high magnitude.

The few studies that have examined underlying population vulnerability have tended to use community level analyses that found that various measures of lower SES will confer greater risk from wildfire smoke [15,19,31,46,47]. Although a Canadian study did not, this null finding may be related to Canada’s more comprehensive healthcare system [48]. Several studies only detected wildfire health effects in a subgroup with both health and SES vulnerabilities—the indigenous population in Australia—as parallel analyses with the general population failed to detect an effect [48,49]. An analysis of the same San Diego wildfire using Kaiser Permanente health plan members appeared to have possibly lower increases in emergency room visits than our findings, although the analyses are not directly comparable [50]. Our study population of Medi-Cal beneficiaries would encompass multiple susceptibility factors, which may manifest during disasters in ways beyond those directly related to baseline health, e.g., having fewer resources to evacuate, less effective home air filtration, or less control over work schedules.

A limitation of this analysis is that, because Medi-Cal data was used, the study population is not representative of the general population. At the same time, some of the populations most vulnerable to the health effects of wildfires are well-represented among Medi-Cal beneficiaries. For example, over 50% of the state’s aged 0–4 population is covered by Medi-Cal [51]. Children are generally more vulnerable to air pollution due to their higher ventilation rate and other factors [52]. A further limitation may be our use of only fee-for-service claims. In 2007, 48% of San Diego Medi-Cal beneficiaries were in managed care [29], and we have no information on differences between the fee-for-service and managed-care populations that could affect our findings. Medi-Cal data only included a primary and secondary diagnosis code, so any condition not occurring within the first 2 codes would not be identified. There is always a possibility of misclassification in the diagnosis codes or missing data on utilization; however, this should be limited by using medical claims data that are required to be submitted for payment. In addition, the relatively short time frame of this study should reduce any limitations that are a result of changing Medi-Cal eligibility over time.

Our wildfire smoke models allowed geospatially and temporally resolved outputs of particulate concentrations. However, our analysis was based on patient residential zip code, so exposure misclassification would occur because people change location during the day. Wildfire-related disruptions could also have prevented people from seeking care or have caused diversion to facilities outside the area, which would bias our results toward the null. Still, because of the widespread nature of the smoke across much of the populous area of the county, the use of exposure periods defined by sets of wildfire dates appeared to perform relatively well in capturing a broad population risk.

As the population ages and the prevalence of comorbidities increase, the number of persons who are susceptible to wildfire exposures will also grow. Nationally, the proportion of the population over age 65 is anticipated to grow from 15% to 24% by 2060 [53]. Increasing prevalence of diabetes and obesity in the US [54] will also impact cardiovascular health. Unless these trends are reversed, the growing older population will also be less healthy, leading to a greater segment of the population vulnerable to PM from wildfires.

## Summary and conclusions

Our study of Medi-Cal beneficiaries identified a significant increase in adverse respiratory events from wildfire smoke exposure and suggested that health risk may persist beyond several immediate days of high–PM exposure. Our findings contribute to growing evidence that, in addition to acute respiratory events such as asthma exacerbation, exposure to wildfire PM may predict infectious conditions, including upper respiratory infections, bronchitis, and pneumonia, which may take longer to manifest. The substantial risk noted among the youngest children is cause for concern because of the potential for long-term harm to children’s lung development. The vulnerability of our study population was also shown in its sensitive response to deteriorating air quality because excess adverse health events began to occur at mildly degraded levels of air quality.

The risk of future wildfires to the health of Californians will continue to be shaped by global climate change, as well as the characteristics and anticipated growth of vulnerable subpopulations. The recognition that climate change will increase the burden most severely on disadvantaged communities creates the imperative for public health to help prepare and protect these vulnerable populations.

## Supporting information

S1 Protocol IRB Protocol—Committee for the Protection of Human Subjects, 14-08-1679. IRB, International Review Board.
(PDF)

S1 Table Respiratory and cardiovascular emergency department presentations, hospital admissions, and outpatient presentations (RRs) for day 1–5, day 6–10, day 11–15 exposure periods; San Diego County, 2007. RR, rate ratio.
(DOCX)

S2 Table Age-specific RRs for respiratory outcomes for very young children aged 0–1, young children aged 2–4, young children aged 0–4, older children aged 5–17, and adults aged 18–64; October 22–26, 2007, San Diego County. RR, rate ratio.
(DOCX)

S1 Data Excel dataset of daily average wildfire PM_2.5_ by date and zip code, San Diego County, July 11 through December 31, 2007. PM_2.5_, fine inhalable particles that are 2.5 micrometers and smaller.
(XLSX)

S1 RECORD Checklist RECORD checklist. (DOCX)

## Abbreviations

AIC: Akaike information criteria
AQI: Air Quality Index
CI: confidence interval
COPD: chronic obstructive pulmonary disease
DHCS: Department of Health Care Services
HYSPLIT: Hybrid Single-Particle Lagrangian Integrated Trajectories
ICD: International Classification of Diseases
MIS/DSS: Management Information System/Decision Support System
MLE: maximum likelihood estimate
OR: odds ratio
PM: particulate matter
PM_10_: inhalable particles that are 10 micrometers and smaller
PM_2.5_: fine inhalable particles that are 2.5 micrometers and smaller
RR: rate ratio
SES: socioeconomic position and status
US EPA: US Environmental Protection Agency
WFEIS: Wildland Fire Emissions Information System
