# Supplementary material for: The San Diego 2007 wildfires and Medi-Cal emergency department presentations, inpatient hospitalizations, and outpatient visits: An observational study of smoke exposure periods and a bidirectional case-crossover analysis
Source: PLoS Med. 2018 Jul 10;15(7):e1002601. doi: 10.1371/journal.pmed.1002601 (PMC6038982; doi:10.1371/journal.pmed.1002601)
Supplement: S1 Protocol — IRB, International Review Board. (PDF) [file pmed.1002601.s001.pdf]

Personnel Information ... 1

Vulnerable Population Checklist ... 2

Study Location ... 2

General Checklist ... 2

Funding ... 3

Expedited Paragraphs ... 3

Purpose, Study Procedures, Testing of a New Drug or Devices ... 4

Study Affiliation ... 8

Subject Population ... 8

Risks,Benefits ... 10

Data Security Requirements Administrative Safeguards,Physical Safeguards,Electronic Safeguards ... 12

Conflict of Interest ... 16

Informed Consent ... 17

Assent Background ... 17

HIPAA ... 18

Assurance of Consistency ... 19

Attachments ... 20

Obligations ... 20

Event History ... 22

PROTOCOL
Request for CPHS Approval
Committee for the Protection of Human Subjects

Protocol Title: Identifying and describing the spectrum of public health morbidity and health care services costs associated with the 2007 San Diego wildfires within a Medi-Cal population

Protocol Type: Request for CPHS Approval

Date Submitted: 09/05/2014

Approval Period: 10/03/2014-10/02/2015

Important Note: **This Print View may not reflect all comments and contingencies for approval. Please check the comments section of the online protocol. Questions that appear to not have been answered may not have been required for this submission. Please see the system application for more details.**

---

* * * Personnel Information * * *

Principal Investigator (required)

Name
Hoshiko, Sumi

Title
Research Scientist

Credentials
MPH

Email
sumi.hoshiko@cdph.ca.gov

Phone
510-620-3668
510-620-3720

Fax
510-620-3720

Organization Name
California Department of Public Health

Additional Organizational Information

Mailing Address
850 Marina Bay Parkway, Bldg P, 3rd Floor Richmond, CA 94804

Alternate Phone

Training data is not currently needed

Co-Principal Investigator (if applicable)

Name
Hutchinson, Justine

Title
Cal-EIS Fellow

Credentials
PhD, MPH

Email
Justine.Hutchinson3@cdph.ca.gov

Phone
510-231-7647

Fax

Organization Name
California Department of Public Health

Additional Organizational Information

Mailing Address

Alternate Phone

Training data is not currently needed

Responsible Official (required)

Name

Title

Credentials

Protocol Title: Identifying and describing the spectrum of public health morbidity and health care services costs associated with the 2007 San Diego wildfires within a Medi-Cal population

---

Prudhomme, Janice | Chief, Environmental Health Investigations Branch | DO, MPH

Email
janice.prudhomme@cdph.ca.gov

Phone
510-620-3617
510-620-3720

Fax
510-620-3720

Organization Name
California Department of Public Health

Additional Organizational Information

Mailing Address
850 Marina Bay Parkway, Bldg P, 3rd Floor Richmond, CA 94804

Alternate Phone

Training data is not currently needed

---

* * * Vulnerable Population Checklist * * *

Vulnerable Population(s) Checklist

Select All That Apply :

Minors (In the United States, a minor is under 18 years of age. If research is conducted outside the United States, a minor is under the age of majority in the countries where research is to be conducted.)

Prisoners

Pregnant Women, Fetuses, and/or Neonates

X **Not Applicable**

Other

---

* * * Study Location * * *

Study Location(s)

Not applicable

X **Study Location (specify below)**

San Diego County is the study location. Data from LA and Orange counties may also be used for comparison.

---

* * * General Checklist * * *

Project Type

Death-Data Only

Protocol Title: Identifying and describing the spectrum of public health morbidity and health care services costs associated with the 2007 San Diego wildfires within a Medi-Cal population

---

SB-13 (Information Practices Act)
X Common Rule

Includes:
X Minimal Risk
Non-English Translation
X HIPAA Waiver
Consent Form
Assent Form
Reliance Agreement Relying on CPHS
Reliance Agreement with Another IRB

---

* * * Funding * * *

Funding Source(s)

X None

---

* * * Expedited Paragraphs * * *

Please check the criteria below that you think your project meets to qualify for an expedited review. If none of these expedited criteria are appropriate for your project, please move to the next screen without selecting any of these criteria; your protocol will be reviewed by the full committee.

New project requesting only previously existing *personally identifiable data or specimens (PIDS) from departments outside of **the California Health and Human Services Area (CHHSA). (Information Practices Act (IPA) review)

New project requesting only previously existing PIDS and not involving state research staff, funding or state mental hospital patients from departments within the CHHSA (IPA review)

X New project requesting only previously existing PIDS involving CHHSA department funding , research staff or patients from state mental hospitals (Common Rule review)

Death data-only projects (Death Data review)

Note that CPHS will make the final determination of whether the project meets the criteria for expedited review.

* PIDS is defined as existing data or specimens including any of the 18 HIPAA identifiers. http://www.oshpd.ca.gov/Boards/CPHS/HIPAAIdentifiers.pdf

**The Departments within the CHHSA are: Aging, Alcohol and Drug Programs, Child Support Services, Community Services and Development, Developmental Services, Emergency Medical Services Authority, Health Care Services, Mental Health, Public Health, Rehabilitation, Social Services, Statewide Health Planning and Development

---

* * * Purpose, Study Procedures, Testing of a New Drug or Devices * * *

Original Project Number (#00-00-00) 14-08-1679

Title (Please indicate if the protocol title is different from the proposal title)

Identifying and describing the spectrum of public health morbidity and health care services costs associated with the 2007 San Diego wildfires within a Medi-Cal population

Start Date: 10/01/2014 **End Date:** 12/31/2015

Complete Sections 1 - 11. Specify N/A as appropriate. Do not leave any sections blank.

1. Purpose of the study

a) **Include a brief statement , less than 500 words, describing the research project. Be sure to address the background for the project, including relevant literature, the major research questions to be addressed, and the expected end product (e.g., article, report or other publications). Include the location(s) where the project will take place. The summary should be understandable to the general public.**

Large-scale California wildfire events are projected to increase through the end of the century (Westerling et al., 2011). Studies have consistently demonstrated increases in respiratory ailments during wildfires, and some studies have also shown cardiovascular effects (Reid, 2014). Most wildfire studies in California have been limited to hospital and emergency department visits, capturing severe morbidity, but likely missing important health burdens. A study of California children found increases in self-reported respiratory and ocular symptoms, medication use, and physician visits when the community was exposed to smoke from wildfires (Künzli et al., 2006). A survey of wildfire evacuees in California found that more persons sought care at a private doctor rather than an emergency department; furthermore, 28% of

---

persons reported leaving prescription medication behind (Jenkins et al., 2009). Canadian researchers found increases in physician visits for respiratory but not cardiovascular outcomes (Moore et al., 2009). More sensitive studies are needed to identify the full spectrum of health sequelae from wildfires in California.

Known as the San Diego Firestorm, a cluster of wildfires burning in San Diego County in fall 2007 resulted in many inhabited areas being subjected to thick smoke and caused a mandatory evacuation of 500,000 residents. To understand the burden of large wildfire events on public health and the health care system, we are asking: What health impacts are discernable from the outpatient visits, emergency and urgent care visits, and hospitalizations of MediCal patients who lived within the exposure area? What is the magnitude of additional disease burden in terms of excess health care visits, and what types of health conditions are affected? What vulnerable subpopulations exist as identified by demographic characteristics? And what is the estimated cost to the MediCal Program and patients of additional outpatient, emergency and urgent care visits and hospitalizations?

Collaborating wildfire experts at the Michigan Tech Research Institute will provide daily average particulate matter levels by zip code for San Diego for the Firestorm period and comparison periods based on a previous study they conducted on that fire event. We intend to use Department of Health Care Services (DHCS) MediCal claims data from to examine patient visits during the Firestorm period and compare with numbers and types of visits during comparable time periods and locations. We will seek to identify increases in specific diagnostic codes and clusters of related codes during the exposure and lag periods, and estimate increased health care costs associated with the wildfire event.

Research findings will be submitted to a peer-reviewed environmental health journal.

References.
1. Westerling, A. L., B. P. Bryant, H. K. Preisler, T. P. Holmes, H. G. Hidalgo, T. Das, and S. R. Shrestha. "Climate change and growth scenarios for California wildfire." Climatic Change 109, no. 1 (2011): 445-463.
2. Reid, C. BC Centre for Disease Control, (2014). Consensus guidelines for public health decision making during wildfire smoke events evidence review: Health effects of wildfire smoke exposure
3. Künzli N, Avol E, Wu J, Gauderman WJ, Rappaport E, Millstein J, et al. Health effects of the 2003 Southern California wildfires on children. Am J Respir Crit Care Med. 2006;174:1221-8.
4. Jenkins JL; Hsu EB; Sauer LM; Hsieh YH; Kirsch TD. Prevalence of Unmet Health Care needs and description of health care-seeking behavior among displaced people after the 2007 California wildfires. Disaster Med Public Health Prep. 2009; 3(2 Suppl):S24-8.
5. Moore D, Copes R, Fisk R, Joy R, Chan K, Brauer M. Population health effects of air quality changes due to forest fires in British Columbia in 2003: estimates from physician-visit billing data. Can J Public Health. 2006;97:105-8.

b) **What is the major research question to be addressed in this project?**

What were the health impacts of the 2007 San Diego Firestorm on the MediCal population?

2. Study Procedures

a) **Describe all study procedures. Please note: The box below is for text only. If you would like to add tables, charts, etc., attach those files in the Attachment section.**

This study will examine the public health impact of wildfires by examining health conditions associated with outpatient, hospitalization, and emergency department visits during the 2007 San Diego Firestorm, when a cluster of wildfires burned nearly 1 million acres in the county between October 20 and November 9. This

fire event was selected because the exposures have been previously well-characterized (Thelen et al., 2013).

POPULATION IDENTIFICATION AND TIME PERIODS
All Medi-Cal claims records defined by the identified fall 2007 wildfire period (and comparison periods) and location (patient zip code) will be obtained for San Diego County from DHCS. (A secondary analysis may be performed using provider address as a surrogate for patient location as a validation/sensitivity analysis.)

We have requested from DHCS MediCal claims data from San Diego County during the Firestorm period and a lag period, plus, for comparison, those in the months before the fires and those from comparable periods in nearby years (2005, 2006, 2008, 2009). MediCal data from Los Angeles and Orange County have also been requested and will be evaluated for suitability as an alternative comparison. The geographic and temporal area of interest will be based on previous research for this wildfire; specifically, average daily levels of particulate matter levels by ZIP code.

DATA ANALYSIS

DESCRIPTIVE CHARACTERISTICS OF THE STUDY POPULATIONS:
We will describe the population by demographics (age, race, sex) based on the total encounters (visits) dataset, as well as by the population of unique individuals.

EVALUATION OF EXCESS TOTAL OUTPATIENT VISITS

Total excess visits: To determine whether there was an excess number of outpatient visits associated with the wildfire period, we will examine rate ratios for number of total outpatient visits during the wildfire dates vs. the expected number of visits, based on a reference period. The reference (comparison) period will be used to compare numbers of visits in the wildfire exposure dates vs. unexposed time periods. To estimate the expected number of outpatient visits for the wildfire period, we will use an equivalent set of days close in time to the wildfire dates in the same year, with the same distribution of days of week.

The calculation of rate ratios would ordinarily involve denominators of person-time, but on the assumption that the population size remains constant during these months, calculations simplify to outpatient visits per day in the wildfire period divided by visits per day in the referent period. We will calculate confidence intervals using large sample statistics for person-time rate ratios [Rothman, Greenland and Lash, 2008]. Several lag periods will be explored, based on previous literature.

To test whether the reference period may have been unrepresentative and thus biased, we will also conduct the analysis using several alternate reference periods, adjusting rate ratios to avoid possible confounding by day of week by stratifying using the Mantel- Haenszel method. Alternate comparison periods will be drawn from: 1) the same periods for neighboring years (2005, 2006, 2008, 2009), excluding any identified fire periods using the same criteria and exposure data source used to identify the fire study period; and 2) possibly other counties (Orange and Los Angeles), provided that adequate data can be obtained to reliably identify and exclude fire periods/locations and if populations can be shown to be demographically comparable.

Total excess patients accessing services: We will also identify repeat visits by the same individual in order to estimate the increase in total persons accessing outpatient services, in addition to our calculation of total outpatient encounters.

SCREENING FOR INCREASED SPECIFIC AND GROUPS OF HEALTH OUTCOMES
All patient visits (outpatient, urgent care, emergency department and hospitalization) by diagnosis code will be statistically screened to identify those codes that are increased. Estimates of expected numbers of diagnostic codes will be generated using the records from unexposed time periods and locations.

---

diagnostic codes will be generated using the records from unexposed time periods and locations. Statistical approaches to adjust significance testing for multiple endpoints simultaneously (the rate ratios of diagnostic codes) will be employed, e.g. empirical Bayes techniques for screening multiple endpoints [Bender and Lange, 2001; Greenland and Robins, 1991]. These screening techniques will be used to identify outcomes with sufficient evidence to merit further consideration, allowing elimination of those with small numbers which would render their results statistically unstable. Further qualitative evaluation of any elevated endpoints in the screening step would be performed to characterize outcomes and make assessments based on biological and circumstantial plausibility of which may be meaningfully related to wildfire and evacuation impacts. Disease outcomes by category will also be evaluated using ICD-9 groupings in order to identify broader disease categories that may be impacted. ICD-9 groupings may be based on within existing ICD-9 hierarchical classification structure, published literature, or developed empirically from medically related diagnoses that co-occur in the same patient/visits in the unexposed portion of the dataset.

FOCUSED EVALUATION OF RESPIRATORY AND CARDIOVASCULAR OUTCOMES
Focused evaluation of a priori identified respiratory (ICD9 460-519) and circulatory (390-459) outcome groups will be conducted to examine more closely which specific health conditions may be contributing relatively more to excess morbidity in each of these areas.

EVALUATION OF COST BURDEN OF EXCESS MORBIDITY
We will calculate the additional cost burden associated with any increases in total visits during the wildfire period by summing the total costs of visits (based on billed and paid claims variables) in the wildfire period compared to the reference period. We will calculate cost totals of key diagnoses to identify those that most drive costs.

REFERENCES
1. Thelen B, French NH, Koziol BW, Billmire M, Owen RC, Johnson J, Ginsberg M, Loboda T, Wu S. Modeling acute respiratory illness during the 2007 San Diego wildland fires using a coupled emissions-transport system and generalized additive modeling. Environ Health. 2013;12:94.
2. Rothman, K.J., Greenland, S., & Lash, T.L. (2008). Modern Epidemiology, 3rd Edition. Philadelphia, PA: Lippincott, Williams & Wilkins.
3. Bender R, Lange S. Adjusting for multiple testing—when and how? Journal of Clinical Epidemiology. 2001;54(4):343-9
4. Greenland S, Robins J. Empirical-Bayes adjustments for multiple comparisons are sometimes useful. Epidemiology 1991;2:244-51.

b) **State if audio or video taping will occur. Describe how the tapes will be maintained during and upon completion of the project. Describe what will become of the tapes after use (e.g., shown at scientific meetings, erased, etc.).**

No audio or video taping will occur.

c) **State if deception will be used. If so, provide a rationale and describe debriefing procedures. Submit a debriefing script in the Attachment section.**

No deception will be used.

3. Testing of a New Drug or Devices

a) **Is a new drug or device being tested or used in the research project? If yes, attach copies of any state and/or federal documents that permit the investigator(s) to proceed with research.** N

---

b) **Describe the procedures, such as use of a data monitoring committee, to be used for adequately monitoring the safety of the subjects involved in testing a new drug or device.**

---

* * * Study Affiliation * * *

4. Study Affiliation

a) **If data or specimens from departments within the State of California are being requested, list the department name and the formal name of the data base or specimen registry.**

A request for a custom dataset from DHCS has been approved by the Committee at their August 28 meeting, and will be released to us following their departmental approval and CPHS approval. The data bases involved are the 35' Paid Claims File and the Meds Eligibility File.

b) **List any California Health and Human Services Agency department(s)' involvement in providing research staff, funding and/or patients from State mental hospitals for this project.**

CDPH is providing research staff. No State mental hospitals or other CHHSA departments will be providing staff, funding or patients.

---

* * * Subject Population * * *

5. Subject Population

In the space below, please describe the participants that you are requesting to recruit (include requested participant number and description of each group requested).For data-only studies ,describe the databases or records to be accessed and the data elements to be obtained.

a) **Provide a full description of how human subjects will be involved in the research. Address characteristics of subjects, such as age, sex and ethnicity and number of participants.**

This study uses existing health data; no subjects will be contacted for this study. The subjects are those Medi-Cal beneficiaries in San Diego County who had a medical visit during the study period that qualified them for inclusion in the study. The demographic characterization of the subjects will be done as part of the study.

Information is available regarding the Medi-Cal population in San Diego County during Fiscal Year 2007-2008. There were a total of 367,799 Medi-Cal beneficiaries, constituting 11.6% of the total population of San Diego County. 58% of beneficiaries were female and 42% were male. 50% were ages 18 and below (25% female, 25% male); 23% were ages 19-44 (17% female, 7% male); 7% were ages 45-54 (4% female,

---

3% male); 5% were ages 55-64 (3% female, 2% male); 15% were ages 65 and above (9% female, 5% male). By language, 51% spoke primarily English and 33% spoke primarily Spanish. Race/ethnicity data for the adult population is not available. The racial/ethnic make-up of the San Diego County Medi-Cal youth population (under 18 years of age) in FY2007-2008 was: 59% Hispanic, 14% White, 10% African American, 6% Asian/Pacific Islander, <1% Native American and 10% Other/Unknown. The distribution of ages of the youth population was: 41% 0-5 years; 30% 6-11 years and 39% 12-17 years.

Los Angeles and Orange Counties will be evaluated for suitability as sources for comparison periods.
In Los Angeles County, the FY2007-2008 Medi-Cal beneficiary population was 57% female and 43% male. 50% were ages 0-18; 25% were ages 19-44; 6% were ages 45-54; 4% were ages 55-64; and 14% were ages 65 and above. By language, 41% spoke primarily English, 47% spoke primarily Spanish, and 13% spoke some other language.
In Orange County, the FY2007-2008 Medi-Cal beneficiary population was 57% female and 43% male. 51% were ages 0-18; 24% were ages 19-44; 6% were ages 45-54; 4% were ages 55-64; and 15% were ages 65 and above. By language, 38% spoke primarily English, 44% spoke primarily Spanish, and 19% spoke some other language.

References:
1. State of California, Department of Health Care Services. Proportion of Population Enrolled by County, July 2008. Report Date: April 2010. Available at: http://www.dhcs.ca.gov/dataandstats/statistics/Documents/18_Medi_Cal_population_by_County_2008.pdf
2. State of California, Department of Health Care Services. Beneficiaries by Age and Gender By County, July 2008. Report Date: April 2010. Available at: http://www.dhcs.ca.gov/dataandstats/statistics/Documents/18_Medi_Cal_population_by_County_2008.pdf
3. State of California, Department of Health Care Services. Language By County, July 2008. Report Date: April 2010. Available at: http://www.dhcs.ca.gov/dataandstats/statistics/Documents/18_Medi_Cal_population_by_County_2008.pdf
4. County of San Diego Health & Human Services Agency, Child and Adolescent Services Research Center (CASRC). Children's Mental Health Annual System of Care Report – FY2007-2008. Report Date: December 3, 2009. Available at: http://www.sdcounty.ca.gov/hhsa/programs/bhs/documents/CMHS0708.pdf

b) If existing data will be obtained, list the database(s) to be used, the time period(s) being requested. This may include requests for future data that is not available at this time. List or attach a list of variables (in the Attachment Section, Attachments) being requested and justify the need for each variable and for the quantity of data being requested. Also, will participants be involved in any other studies?

A request for a custom dataset from DHCS has been approved by the Committee at their August 28 meeting, and will be released to us following their departmental approval and CPHS approval (from the DHCS 35' Paid Claims File and the Meds Eligibility File).

We are requesting data that encompass unexposed time to generate stable estimates of expected rates, the exposure time (study period), and a lag period immediately following the exposure period. We are also requesting data for alternate unexposed periods. The periods requested are the months of August-December for the years 2005-2009.

Please see attached Data Table for a list of variables requested and justifications for each variable. Study subjects will not be involved in any other studies.

c) What is the rationale for studying the requested group(s) of participants?

Protocol Title: Identifying and describing the spectrum of public health morbidity and health care services costs associated with the 2007 San Diego wildfires within a Medi-Cal population

---

We chose to study a large population impacted by the 2007 San Diego Firestorm. The magnitude of the fire event makes it desirable as a basis for this analysis as its greater sample size will provide an excellent opportunity to identify wildfire-related health conditions.

Further, we have access to extensive exposure information that would allow us to effectively define our targeted study group and comparison populations.

The MediCal dataset is also advantageous in that it contains data on outpatient visits, information that is not readily elsewhere available (e.g. it is not part of the data available through the Office of Statewide Health Planning and Development, which only captures hospital admissions and emergency visits and some ambulatory care.) The dataset will also allow study of impacts within a relatively vulnerable population, as the MediCal population is largely comprised of children, elderly, adults with disabilities, and those with fewer resources.

d) Describe how potential subjects will be identified for recruitment. Examples include: class rosters, group membership, individuals answering an advertisement, organization position titles (i.e., Presidents, web designers, etc.). How will potential participants learn about the research and how will they be recruited (e.g., flyer, email, web posting, telephone, etc.)? Attach recruitment materials in the Attachment Section, Attachments. Important to remember: subjects cannot be contacted before IRB approval.

Not applicable; there is no recruitment for this study.

Will you be using recruitment materials ? N

e) Screening Procedures: If subjects will be screened prior to entry into the research, please address the criteria for exclusion and inclusion in the research. Provide reasons for not including women or minorities. Provide justification for including vulnerable populations such as children or prisoners. Please also provide a statement regarding what will happen to the information collected about the individual should they not enter into the study.

Not applicable; there are no screening procedures in this study. All MediCal beneficiaries will be included.

f) Explain the amount, nature, e.g.; gift card, cash, and schedule of compensation, if any, that will be paid for participation in the study. Include provisions for prorating payment. The amount should not be coercive.

Not applicable; no compensation will be paid to subjects.

g) Estimate the probable duration of the entire study. This estimate should include the total time each subject is to be involved and the duration the data about the subject is to be collected (e.g., This is a 2-year study. Participants will be interviewed 3 times per year; each interview will last approximately 2 hours. Total approximate time commitment for participants is 12 hours.)

This is a data only study; subjects will not be contacted. The analysis will be performed from September or October 2014 (pending receipt of data and CPHS approval) to December 2015.

---

* * * Risks,Benefits * * *

Protocol Title: Identifying and describing the spectrum of public health morbidity and health care services costs associated with the 2007 San Diego wildfires within a Medi-Cal population

---

6. Risks (Input N/A if not applicable)

US Department of Health & Human Services (HHS) Regulations define a subject at risk as follows: "...any individual who may be exposed to the possibility of injury, including physical, psychological, or social injury, as a consequence of participation as a subject in any research, development, or related activity which departs from the application of those accepted methods necessary to meet his needs, or which increases the ordinary risks of daily life, including the recognized risks inherent in a chosen occupation or field of service."

a) **Provide description of risk, physical, psychological, social or economic, loss of data security and confidentiality to subjects. Describe and justify risk level (minimal, moderate or high).**

Risks in this study are minimal. Patient names and addresses will not be part of the dataset, and the study will summarize large amounts of data in aggregate. However, the databases have zip code, dates of service, and age, and there is some risk of breach of confidentiality for these records (albeit without names or addresses). This possibility will be addressed in the following section on handling in accordance with CDPH Information Security Policies.

b) **If death data is being used, include risk to estate of deceased or living person by use of the death data.**

Not applicable.

c) **If audio/video taping will be used, state if it could increase potential risk to subject's confidentiality.**

Not applicable.

d) **Describe how medical services will be provided if subjects suffer adverse mental or physical effects as result of research activity. If no services provided state clearly.**

Not applicable.

e) **In the case of overseas research, describe qualifications/preparations that enable you to evaluate cultural appropriateness and estimate/minimize risks to subjects.**

Not applicable.

f) **Describe any less risky methods and why they are not being used.**

Less risky methods are not available.

7. Benefits

a) **Benefits: Describe the benefits, if any, to the subjects or to society that will be realized as a result of this project. Discuss the benefits that may accrue directly to the subjects as well as to society. If there is no direct benefit anticipated for the subjects, please state such.**

There are no direct benefits to individual subjects in this data-only analysis.
Indirect benefits to the California population include: better understanding of the health effects and impacts on different types of health services will enhance prevention and mitigation of adverse health outcomes; inform development of syndromic surveillance systems; and help public health administrators in preparedness planning to achieve more effective responses to future large scale wildfire events. The benefits to the MediCal program are described in the attached DHCS application (Section V).

Protocol Title: Identifying and describing the spectrum of public health morbidity and health care services costs associated with the 2007 San Diego wildfires within a Medi-Cal population

---

b) **Explain why study risks are reasonable in relation to the potential benefits to subjects and to society.**

Risks to subjects are minimal. Understanding the health impacts of large wildfire events and characterizing vulnerable groups is essential for developing effective public health interventions during large wildfire events.

*** * * Data Security Requirements Administrative Safeguards,Physical Safeguards,Electronic Safeguards * * ***

8. Administrative Safeguards

a) **Describe the procedures for training all research staff, who have access to PID on privacy and security. Indicate if staff are required to sign a confidentiality statement related to general use, security and privacy**

All staff will receive training through the Environmental Health Investigations Branch (EHIB) on the confidentiality protocols for the Branch and specifically as applied to this project. EHIB has developed and follows a branch-specific confidentiality policy, available at: http://www.ehib.org/cma/topic.jsp?topic_key=136. Staff must read and sign an Oath of Confidentiality regarding procedures for handling confidential materials and data files.
All staff with access to PID for this project will have also completed the Information Privacy and Security Training modules required of California Department of Public Health employees.

b) **Describe procedures, either background check or thorough reference check, for vetting staff, who will have access to PID.**

All staff who will have access to PID are required to undergo the training described above. No additional background checks are conducted.

c) **Indicate whether you have obtained and submitted to CPHS a statement from the state agency or department you are receiving data from. That statement should include that the release of the desired data is legal and that the entity is willing to release the desired data to you, the researcher.**

A request for a custom dataset from DHCS was approved by the Committee at their August 28 meeting, and will be released to us following their departmental approval and CPHS approval. DHCS informed us Sept 4 by telephone that our request was approved and that we will receive a written statement confirming the approval for release.

d) **Explain how you will ensure that data will not be reused or provided to any unauthorized person or entity (unauthorized means that the person or entity does not have a need to access the data for purposes of the research project approved by CPHS?**

To prevent unauthorized data capture, project data shared between researchers will not be accessible through the Internet. Staff will transmit PID between researchers and participating institutions by using encrypted CDs or encrypted email. Data that does not contain PID will be shared only with other authorized staff via email or encrypted CD.

e) **Indicate whether information will not be published that could possibly be used to identify an individual subject.**

No personal health information or other identifying information will be listed in any publication or report produced by this research project. All publicly available data will be aggregated.

f) **Provide adequate justifications for the quantity of the data, the years and the variables being requested. Have you requested no more than the minimum necessary data to perform the research?**

The minimum required data was requested from DHCS. Time periods requested were selected based on the time of the exposure being studied, the potential for a lag period, and the need for unexposed comparison periods, including remote unexposed periods. Justifications for each variable requested are provided in the attached Data Elements sheet.

g) Indicate if access to data limited only to those with a need to know for purposes of implementing or evaluating the research.

Access to the data for this study will be limited to only those project staff who will be analyzing the data and writing reports.

h) If applicable, justify why unique identifiers, other than social security numbers, cannot be used.

N/A. No social security numbers will be used at any time in this study. Unique identifiers called Beneficiary ID Numbers and NOT social security numbers will be provided by DHCS with the data set.

i) Described appropriate and sufficient methods to protect the identity of individual subjects when small cells or small numbers and/or data linkage to another data set are involved in the research project.

If we have a table with an intersection cell that contains a small number of participants (n=1-15), then we will combine rows or columns with adjacent data to increase the cell size. Within narrative reports, whenever the project describes 1-15 subjects, project staff will be especially careful that the identifiers of all subjects are protected from possible disclosure.

j) If the data set is to be linked with any other data sets, identify all data sets and each of the variables to be linked, with justification for each linkage. If there is an extensive list, include the list as an attachment, in the Attachment Section.

N/A

k) If a third party is being used to perform data matching, provided evidence of the third parties' ability to protect PID, including third parties' ability to comply with all the CPHS data security standards

N/A

l) Indicate that you will provide CPHS with a letter certifying that PID has been destroyed and/or returned to the data source once research is concluded.

We will provide CPHS with a letter certifying that PID has been destroyed once the research has been concluded.

m) Include a certification from the Chief Information Officer, Privacy Officer, Security Officer or equivalent position of the researcher's institution that CPHS Data Security Standards are met.A letter or statement assuring these standards are met from this individual on organizational letterhead may be included as an attachments in the Attachment Section.

CDPH's Information Security Officer (ISO) and the Department Privacy Officer are jointly responsible for maintaining data security standards that are equivalent to CPHS Data Security Standards. See the CDPH Health Administrative Manual on information technology services, available at http://www.cdph.ca.gov/programs/DFDRS/Documents/HAM%20Section%206-1000%20Revised%20Aug%2009.pdf

9. Physical Safeguards

a) Indicate that research records and physical samples will be protected through the use of locked cabinets and locked rooms; PID in paper form will not be left unattended unless locked in a file cabinet, file room ,

---

and locked rooms; PID in paper form will not be left unattended unless locked in a file cabinet, file room , desk, or office.

PID and all study materials containing data in paper format are not left unattended in the workplace. These items are stored in a locked file cabinet within a secure room with very limited access.

b) **State whether data/samples will be destroyed or returned as soon as it is no longer needed for the research project.**

Data will be destroyed as soon as it is no longer needed for the research project. No samples will be collected for this study.

c) **If samples are to be retained, will they have personal identifies or be de-identified?**

No samples will be collected in this study.

d) **Describe how you will ensure the PID in paper form is disposed of through confidential means, such as cross cut shredding or pulverizing.**

PID in paper form will be destroyed through cross-cut shredding onsite by study staff or securely delivered to a commercial entity specializing in destruction of confidential data.

e) **Describe how you will ensure that faxes with PID are not left unattended and fax machines are in secure areas.**

No PID in this study is transmitted by fax.

f) **Indicate whether mailings of PID are sealed and secured from inappropriate viewing; mailings of 500 or more individually identifiable records of PID in a single package, and all mailings of PID to vendors/contractors/co-researchers are sent using a tracked mailing method, which includes verification of delivery and receipt, such as UPS, U.S. Express Mail, or Federal Express, or by bonded courier.**

No PID in this study is transmitted by mail.

g) **State whether PID in paper or electronic form, e.g., stored on laptop computers and portable electronic storage media (e.g., USB drives and CDs), will never be left unattended in cars or other unsecured locations. .**

PID in paper or electronic format will never be left unattended in cars or other unsecured locations.

h) **Describe whether facilities, which store PID in paper or electronic form, have controlled access procedures, and 24 hour guard or monitored alarm service.**

PID in paper format is secured in a locked file cabinet within a locked room with controlled access in a building that also has keycard controlled access and 24-hour/7days-per-week security guard surveillance. PID in electronic format is secured on computers that are password protected within the same keycard controlled building with 24-hour/7days per week security guard surveillance as described above.

i) **Provide a description of whether all servers containing unencrypted PID are housed in a secure room with controlled access procedures.**

All servers containing either encrypted or unencrypted PID are housed in secured rooms on the CDPH Richmond campus.

j) **Indicate whether identifiers will be stored separately from analysis data.**

Personal identifiers will be stored in a separate database from those used to analyze the non-PID data..

k) **State whether all disks with PID will be destroyed.**

Yes, all disks with PID will be destroyed.

Protocol Title: Identifying and describing the spectrum of public health morbidity and health care services costs associated with the 2007 San Diego wildfires within a Medi-Cal population

---

10. Electronic Safeguards

a) State whether all computer access be protected through the use of encryption, passwords, and other protections.

Yes, all computers that will be used to evaluate and work with this data are password-protected. Files with PID are also protected and can only be accessed by authorized project staff.

b) Indicate whether all workstations that contain PID have full disc encryption that uses FIPS 140-2 compliant software.

Symantec Endpoint Protection Release 12.1 RU 1 provides the Symantec Cryptographic Module cryptography library modules to protect server-to-server communication and console-to-server communication. It provides the Microsoft FIPS modules to protect client-to-server communication.

c) Indicate if all laptops that contain PID have full disc encryption that uses FIPS 140-2 compliant software

No laptops will contain PID at any time.

d) Note if PID on removable media devices (e.g. USB thumb drives, CD/DVD, smartphones, backup tapes) are encrypted with software which is FIPS 140-2 compliant.

No PID will be stored or transported on thumb drives, smartphones or tapes. CDs with PID will be protected by Symantec Endpoint Protection Release 12.1 RU 1, which provides the Symantec Cryptographic Module cryptography library modules to protect server-to-server communication and console-to-server communication. It provides the Microsoft FIPS modules to protect client-to-server communication.

e) Indicate if all workstations, laptops and other systems that process and/or store PID have security patches applied in a reasonable time frame.

Yes, all our workstations, servers, and laptops that process or store PID have security patches applied in a reasonable time frame.

f) Indicate if sufficiently strong password controls are in place to protect PID stored on workstations, laptops, servers, and removable media.

Password controls (character type requirements, scheduled password updates) are required for accessing all data, including PID, stored on workstations, laptops, servers and removable media.

g) Indicate if sufficient system security controls are in place for automatic screen timeout, automated audit trails, intrusion detection, anti-virus, and periodic system security/log reviews?

IT staff conduct periodic reviews of our IT security system and logs. Automatic screen timeout, automated audit trails, anti-virus and intrusion detection are all provided for each workstation and all servers.

h) Explain whether all transmissions of electronic PID outside the secure internal network (e.g., emails, website access, and file transfer) are encrypted using software which is compliant with FIPS 140-2.

All transmission of electronic PID outside the secure CDPH network utilize Symantec Endpoint Protection Release 12.1 RU 1 which provides the Symantec Cryptographic Module cryptography library modules to protect server-to-server communication and console-to-server communication. It provides the Microsoft FIPS modules to protect client-to-server communication.

Protocol Title: Identifying and describing the spectrum of public health morbidity and health care services costs associated with the 2007 San Diego wildfires within a Medi-Cal population

---

i) Note if PID in an electronic form will be accessible to the internet.

Encrypted PID is not accessible to an uncredentialized user on the internet.

j) When disposing of electronic PID, indicate whether sufficiently secure wiping, degaussing, or physical destruction will be used

To dispose of electronic PID CDPH uses GDisk or Autoclave to completely wipe hard drives and data from all workstations, laptops and servers.

---

* * * Conflict of Interest * * *

11. Conflict of Interest

Describe any financial or other relationships of the researcher(s) or the institution that could be perceived as affecting the objective conduct of the research, including the interpretation and publication of the findings.

Financial relationships to be disclosed include but are not limited to the following:

* Present or anticipated ownership of stock, stock options, or other financial obligations of the source of funding.
* Receipt or expectation of payment of any sort in connections with papers, symposia, consulting, editing, etc. from the source of funding.
* The sale or licensing or anticipated sale or licensing of medical or other products or intellectual property, such as patents, copyrights, or trade secrets to the source of funding or other entities.
* Any past, present or anticipated receipt of money or other valuable consideration from the source of research funding by the researcher(s), the family of the researcher(s), the research institution, or by an institution in which the researcher(s) or the family of the researcher(s) has an interest as owner, creditor, or officer.

Does any member of the study team, members' spouses, or members' dependent children have any significant financial interests related to the work to be conducted as part of the above-referenced project? N

Name of Personnel with Financial Conflict of Interest

Other research staff that may have a conflict. Please specify below.

Protocol Title: Identifying and describing the spectrum of public health morbidity and health care services costs associated with the 2007 San Diego wildfires within a Medi-Cal population

---

Any member of the study team who answers in the affirmative must be listed in the box below.

A staff person will contact any researcher listed above to obtain additional information regarding the specific financial interest(s).

I certify that all members of the study team have answered the financial interests question and only those individuals listed in the box above have disclosed any financial interest related to this study. Y

---

* * * Informed Consent * * *

12. Informed Consent

Provide a description of procedures to be used in obtaining and documenting the prior informed consent of participants. Further CPHS instructions and consent format may be found on the CPHS website link: http://www.oshpd.ca.gov/Boards/CPHS/index.html.

Not applicable.

Non-English versions of consent/assent forms or scripts must be submitted as attachments in the Attachment section , along with the curriculum vitae of the translators(s) and/or proof of certification of the translation firm. CPHS may reject poorly written documents or documents from translators lacking adequate proof of training or expertise. In general, Spanish translation should use formal language.

CPHS may approve the use of a consent procedure which does not include, or which alters, some or all of the elements of informed consent. If a waiver or alteration of informed consent is being requested, provide information as to how all of the criteria below will be satisfied.

Informed Consent

Title	Consent Type	Create Date
Blank Page	Documentation of Consent	09/05/2014

Attachments Section

---

* * * Assent Background * * *

13. Assent Background

Protocol Title: Identifying and describing the spectrum of public health morbidity and health care services costs associated with the 2007 San Diego wildfires within a Medi-Cal population

---

Provide a description of procedures to be used in obtaining and documenting the prior assent of participants. Further CPHS instructions and assent format may be found on the CPHS website link: http://www.oshpd.ca.gov/Boards/CPHS/index.html.

Not applicable.

While the intent of informed assent is the same as that of informed consent, the informed assent must be written at a level that is understandable to potential participants who are children between the age of 7-17 years of age. Different informed assent forms may be needed if the study involves children of significantly different ages. The same headings must be used. Because some children cannot read through as long a form as an adult, assent forms may be shortened to facilitate reading and understanding by children. However, all of the required elements of the informed consent must still be adequately addressed. The CPHS website link to the format and additional instructions is : http://www.oshpd.ca.gov/Boards/CPHS/InstructionsforResearchers.pdf#Untitled (53).

See sample assent/consent forms at http://www.keyusa.com/human-subjects.html
If applicable, provide assent process background information for each Assent Form, Alteration of Assent Form (i.e., Cover Letter or Verbal Script), or Waiver.

Assent Background

Title	Assent Information Type	Create Date
Blank Page	Documentation of Assent	09/05/2014

Attachments Section

---

* * * HIPAA * * *

14. Health Insurance Portability Accountability Act (HIPAA)

To determine if data for this project is covered by HIPAA, respond to the four questions below.

1. Will health information be obtained from a covered entity, known as a clearinghouse, such as Blue Cross, that processes or facilitates processing health data from another entity, including but not limited to state databases? Y

2. Will the study involve the provision of healthcare by a covered entity, such as the UCD Medical Center? N

3. If the study involves the provision of healthcare, will a health insurer or billing agency be contacted for billing or eligibility? N

---

4. Will the study involve other HIPAA criteria not listed above? N

If you answered "YES" to any of the questions above, you are subject to HIPAA and must complete a HIPAA authorization or waiver request with your protocol.

Use table below ONLY when requesting waiver/alteration of HIPAA authorization.

HIPAA

Title	HIPAA Information Waiver Type	Attached Date
Identifying and describing the spectrum of public health morbidity and health care services costs associated with the 2007 San Diego wildfires within a Medi-Cal population	Waiver/Alteration of Authorization	

Attachments Section

---

* * * Assurance of Consistency * * *

15. Assurance of Consistency between Grant Application and CPHS Protocol

Is this project funded by a grant? N

If 'Yes' is checked, please attach only the sections of the grant application, in Attachments, that address the questions below. List the page(s) in the grant application that confirm the consistency with the protocol. Also include that section, such as Subject Population, in the Protocol Information that confirms the consistency with the grant application. This section does not apply to contracts.

a) The title of the project in the grant application and the research protocol are the same. If not explain why.

b) The specific aims of the project in the grant application and the research protocol are the same. If not, explain why. Please include the page number(s) where this information may be found on both documents.

c) The research design/methods are the same in the grant application and the research protocol. If not, explain why.Please include the page number(s) were this information may be found on both documents.

Protocol Title: Identifying and describing the spectrum of public health morbidity and health care services costs associated with the 2007 San Diego wildfires within a Medi-Cal population

---

d) The inclusion criteria are the same in the grant application and the research protocol. If not, explain why. Please include the page number(s) were this information may be found on both documents.

e) The human subjects protections, including vulnerable populations, are the same in the grant application and the research protocol. If not, explain why. Please include the page number(s) were this information may be found on both documents.

---

* * * Attachments * * *

Attach all documents associated with your project.

Document Type	Document Name	Attached Date	Submitted Date
Budget	Wildfire_budget_14-08-1679	08/28/2014	09/05/2014
Cover Letter	IRB_wildfire_Cover Letter_14-08-1679	08/28/2014	09/05/2014
Curriculum Vitae of Principal Investigator	IRB_wildfire_HoshikoCV_8-26-2014_Project14-08-1679	08/28/2014	09/05/2014
Curriculum Vitae of the Co-Principal Investigator	IRB_wildfires_Hutchinson CV_14-08-1679	08/28/2014	09/05/2014
Data Security Requirements Letter	IRB_wildfire_Security_Letter_04-08-1679	08/28/2014	09/05/2014
Data Security Requirements Letter	IRB_wildfire_Securitylet_JP_14-08-1679	08/28/2014	09/05/2014
Any Other Documents (title file clearly)	DHCS Data Request Hoshiko 14-08-1679	09/05/2014	09/05/2014
List of study variables	Variables requested Hoshiko 14-08-1679	09/05/2014	09/05/2014
New Project Checklist	New Project Checklist Hoshiko_14-08-1679	09/05/2014	09/05/2014

---

* * * Obligations * * *

Obligations (Researcher's Responsibilities)

The Principal Investigator is ultimately responsible for the conduct of the project. Obligations of the Principal Investigator include:

Conduct the research involving human subjects as presented in the protocol, including modifications, as approved by the Department and Institutional Review Board. Changes in any aspect of the study (for example project design, procedures, consent forms, advertising materials, additional key personnel or subject population) will be submitted to the IRB for approval before instituting the changes (PI will submit the "Amendment/Revision" form);

Provide all subjects a copy of the signed consent form, if applicable. Investigators are required to retain signed consent documents for three (3) years after close of the study;

Submit either the "Protocol Deviation Form" or the "Report Form" to report protocol Deviations/Violations, Unanticipated Problems and Adverse Events that occur in the course of the protocol. Any of these events must be reported to the IRB as soon as possible, but not later than five (5) working days;

Submit the "Continuing Review" Form in order to maintain active status of the approved protocol. The form must be submitted annually at least four (4) weeks prior to expiration, five (5) weeks for protocols that require full review. If the protocol is not renewed before expiration, all activities must cease until the protocol has been re-reviewed;

Certify that all members of the study team have answered the financial interests question and only those individuals listed in section 8 above have disclosed any financial interest related to this study.

Adhere to federal, state and OSHPD policies regarding the rights and welfare of human participants participating in this study.

Comply with and be bound by the U.S. DHHS regulations for the protection of human subjects and relevant ethical principles.

Comply with and be bound by all decisions of the California Health and Human Services Agency Committee for the Protection of Human Subjects.

X **The Principal Investigator has read and agrees to abide by the above obligations.**

X **The Co-Principal Investigator has read and agrees to abide by the above obligations.**

Protocol Title: Identifying and describing the spectrum of public health morbidity and health care services costs associated with the 2007 San Diego wildfires within a Medi-Cal population

---

X **The Responsible Official has read and agrees to abide by the above obligations.**

---

* * * Event History * * *

Event History

Date	Status	View Attachments	Letters
08/20/2014	NEW FORM CREATED		
09/05/2014	NEW FORM SUBMITTED	Y	
10/02/2014	NEW FORM PANEL ASSIGNED		
10/02/2014	NEW FORM REVIEWER(S) ASSIGNED		
10/03/2014	NEW FORM APPROVED	Y	Y
08/03/2015	CONTINUING REVIEW 1 FORM CREATED		
08/24/2015	REPORT 1 FORM CREATED		
08/24/2015	REPORT 2 FORM CREATED		
08/31/2015	REPORT 3 FORM CREATED		
08/31/2015	REPORT 3 FORM SUBMITTED	Y	
09/01/2015	REPORT 3 FORM REVIEWER(S) ASSIGNED		
09/03/2015	CONTINUING REVIEW 1 FORM SUBMITTED	Y	
09/08/2015	CONTINUING REVIEW 1 FORM REVIEWER(S) ASSIGNED		
09/18/2015	CONTINUING REVIEW 1 FORM REVIEWER(S) ASSIGNED		
09/18/2015	CONTINUING REVIEW 1 FORM REVIEWER(S) ASSIGNED		
09/21/2015	CONTINUING REVIEW 1 FORM SUBMITTED (CYCLE 1)	Y	
09/28/2015	REPORT 1 FORM DELETED		
09/28/2015	REPORT 2 FORM DELETED		

10/02/2015	REPORT 3 FORM APPROVED	Y	N
10/05/2015	CONTINUING REVIEW 1 FORM APPROVED	Y	Y
08/08/2016	CONTINUING REVIEW 2 FORM CREATED		
08/30/2016	CONTINUING REVIEW 2 FORM SUBMITTED	Y	
08/31/2016	CONTINUING REVIEW 2 FORM REVIEWER(S) ASSIGNED		
10/07/2016	CONTINUING REVIEW 2 FORM APPROVED	Y	Y
10/07/2017	PROTOCOL EXPIRED		
