# Supplementary material for: The San Diego 2007 wildfires and Medi-Cal emergency department presentations, inpatient hospitalizations, and outpatient visits: An observational study of smoke exposure periods and a bidirectional case-crossover analysis
Source: PLoS Med. 2018 Jul 10;15(7):e1002601. doi: 10.1371/journal.pmed.1002601 (PMC6038982; doi:10.1371/journal.pmed.1002601)
Supplement: S1 Table — RR, rate ratio. (DOCX) [file pmed.1002601.s002.docx]

|  | **Table S1. Respiratory and cardiovascular emergency department presentations, hospital admissions, and outpatient presentations (Rate Ratios) for day 1-5, day 6-10, day 11-15 exposure periods; San Diego County, 2007.** | | | | | | | | | | | | | | | | | | |  |
| --- | --- | --- | --- | --- | --- | --- | --- | --- | --- | --- | --- | --- | --- | --- | --- | --- | --- | --- | --- | --- |
|  | | | **Emergency Department Presentations** | | | | | | | **Hospital Admissions** | | | | | **Outpatient Presentations** | | | | | |
| **Day 1-5** | | | **Exposed** | | **Unexposed** | | **RR** |  | **95% CI** | **Exposed** | **Unexposed** | **RR** |  | **95% CI** | **Exposed** | **Unexposed** | **RR** |  | **95% CI** | |
| Total Episodes of Care | | | 1,107 | | 6,373 | | 1.01 |  | 0.95- 1.08 | 725 | 4,787 | 0.91 | **○** | 0.84- 0.98 | 10,822 | 94,744 | 0.69 | **○** | 0.67- 0.70 | |
| Respiratory Index | | | 298 | | 1,292 | | 1.34 | **●** | 1.18- 1.52 | 148 | 773 | 1.15 |  | 0.96- 1.37 | 1,502 | 8,388 | 1.07 | **●** | 1.02- 1.14 | |
| Asthma | | | 58 | | 164 | | 2.12 | **●** | 1.57- 2.86 | 26 | 101 | 1.54 | **●** | 1.00- 2.38 | 367 | 1,711 | 1.29 | **●** | 1.15- 1.44 | |
| Acute Bronchitis | | | 18 | | 62 | | 1.74 | **●** | 1.03- 2.94 | <10 | 28 | 1.07 |  | 0.37- 2.63 | 110 | 436 | 1.51 | **●** | 1.23- 1.87 | |
| COPD | | | 14 | | 71 | | 1.18 |  | 0.67- 2.10 | 28 | 142 | 1.18 |  | 0.79- 1.77 | 90 | 581 | 0.93 |  | 0.74- 1.16 | |
| Pneumonia | | | 17 | | 77 | | 1.32 |  | 0.78- 2.24 | 34 | 152 | 1.34 |  | 0.93- 1.95 | 57 | 257 | 1.33 |  | 1.00- 1.77 | |
| Upper Respiratory Infection | | | 66 | | 274 | | 1.45 | **●** | 1.10- 1.89 | <10 | 25 | 0.96 |  | 0.29- 2.57 | 715 | 4,353 | 0.99 |  | 0.91- 1.07 | |
| Respiratory Symptoms | | | 189 | | 867 | | 1.31 | **●** | 1.12- 1.53 | 90 | 504 | 1.07 |  | 0.86- 1.34 | 245 | 1,390 | 1.06 |  | 0.92- 1.21 | |
| Bronchitis (not specified) | | | <10 | | 31 | | 1.35 |  | 0.55- 2.97 | <10 | <10 | 2.40 |  | 0.32- 12.17 | 38 | 171 | 1.33 |  | 0.94- 1.89 | |
| Cardiovascular Index | | | 47 | | 297 | | 0.95 |  | 0.70- 1.29 | 88 | 517 | 1.02 |  | 0.81- 1.28 | 168 | 1,527 | 0.66 | **○** | 0.56- 0.77 | |
| Ischemic Heart Disease | | | <10 | | 41 | | 0.88 |  | 0.34- 1.97 | 23 | 147 | 0.94 |  | 0.60- 1.46 | 40 | 403 | 0.60 | **○** | 0.43- 0.82 | |
| Dysrhythmia | | | 17 | | 87 | | 1.17 |  | 0.70- 1.97 | 25 | 121 | 1.24 |  | 0.81- 1.91 | 38 | 277 | 0.82 |  | 0.59- 1.16 | |
| Congestive Heart Failure | | | <10 | | 51 | | 0.71 |  | 0.27- 1.56 | 19 | 126 | 0.90 |  | 0.56- 1.47 | 39 | 358 | 0.65 | **○** | 0.47- 0.91 | |
| Stroke | | | <10 | | 49 | | 1.10 |  | 0.51- 2.17 | 21 | 119 | 1.06 |  | 0.67- 1.68 | 22 | 213 | 0.62 | **○** | 0.40- 0.96 | |
| Diseases of Peripheral Circulation | | | 10 | | 79 | | 0.76 |  | 0.39- 1.47 | 23 | 137 | 1.01 |  | 0.65- 1.57 | 39 | 371 | 0.63 | **○** | 0.45- 0.88 | |
|  | | | | | | | | | | | | | | | | | | | | |
| **Day 6-10** | | | **Exposed** | | **Unexposed** | | **RR** |  | **95% CI** | **Exposed** | **Unexposed** | **RR** |  | **95% CI** | **Exposed** | **Unexposed** | **RR** |  | **95% CI** | |
| Total Episodes of Care | | | 975 | | 6,257 | | 0.93 |  | 0.87- 1.00 | 644 | 4,213 | 0.92 | **○** | 0.84- 1.00 | 10,423 | 58,115 | 1.08 | **●** | 1.05- 1.10 | |
| Respiratory Index | | | 241 | | 1,372 | | 1.05 |  | 0.92- 1.21 | 123 | 704 | 1.05 |  | 0.87- 1.27 | 1,243 | 5,792 | 1.29 | **●** | 1.21- 1.37 | |
| Asthma | | | 41 | | 203 | | 1.21 |  | 0.87- 1.70 | 11 | 95 | 0.69 |  | 0.37- 1.30 | 273 | 1,175 | 1.39 | **●** | 1.22- 1.59 | |
| Acute Bronchitis | | | 13 | | 77 | | 1.01 |  | 0.56- 1.82 | <10 | 17 | 2.12 |  | 0.76- 5.23 | 89 | 311 | 1.72 | **●** | 1.36- 2.17 | |
| COPD | | | 16 | | 81 | | 1.19 |  | 0.69- 2.03 | 23 | 155 | 0.89 |  | 0.57- 1.38 | 78 | 378 | 1.24 |  | 0.97- 1.58 | |
| Pneumonia | | | 18 | | 75 | | 1.44 |  | 0.86- 2.41 | 30 | 153 | 1.18 |  | 0.80- 1.74 | 43 | 196 | 1.32 |  | 0.95- 1.83 | |
| Upper Respiratory Infection | | | 59 | | 324 | | 1.09 |  | 0.83- 1.44 | <10 | 28 | 1.93 |  | 0.86- 4.00 | 594 | 3,136 | 1.14 | **●** | 1.04- 1.24 | |
| Respiratory Symptoms | | | 152 | | 869 | | 1.05 |  | 0.88- 1.25 | 79 | 453 | 1.05 |  | 0.82- 1.33 | 215 | 880 | 1.47 | **●** | 1.26- 1.70 | |
| Bronchitis (not specified) | | | <10 | | 45 | | 0.67 |  | 0.23- 1.57 | <10 | <10 | 0.00 |  | 0.00- 10.29 | 36 | 119 | 1.82 | **●** | 1.25- 2.64 | |
| Cardiovascular Index | | | 46 | | 270 | | 1.02 |  | 0.75- 1.40 | 67 | 504 | 0.80 |  | 0.62- 1.03 | 188 | 942 | 1.20 | **●** | 1.02- 1.40 | |
| Ischemic Heart Disease | | | <10 | | 42 | | 1.29 |  | 0.59- 2.56 | 20 | 142 | 0.85 |  | 0.53- 1.35 | 50 | 252 | 1.19 |  | 0.88- 1.61 | |
| Dysrhythmia | | | 13 | | 84 | | 0.93 |  | 0.52- 1.67 | <10 | 117 | 0.46 | **○** | 0.22- 0.87 | 36 | 170 | 1.27 |  | 0.89- 1.82 | |
| Congestive Heart Failure | | | <10 | | 44 | | 0.82 |  | 0.31- 1.82 | 21 | 127 | 0.99 |  | 0.63- 1.57 | 38 | 209 | 1.09 |  | 0.77- 1.54 | |
| Stroke | | | 11 | | 51 | | 1.29 |  | 0.67- 2.48 | 11 | 124 | 0.53 | **○** | 0.29- 0.99 | 29 | 140 | 1.24 |  | 0.83- 1.85 | |
| Diseases of Peripheral Circulation | | | <10 | | 63 | | 0.76 |  | 0.34- 1.53 | 17 | 130 | 0.78 |  | 0.47- 1.30 | 49 | 236 | 1.25 |  | 0.92- 1.69 | |
|  | | | | | | | | | | | | | | | | | | | | |
| **Day 11-15** | | | **Exposed** | | **Unexposed** | | **RR** |  | **95% CI** | **Exposed** | **Unexposed** | **RR** |  | **95% CI** | **Exposed** | **Unexposed** | **RR** |  | **95% CI** | |
| Total Episodes of Care | | | 939 | | 6,201 | | 0.91 | **○** | 0.85- 0.97 | 676 | 4,060 | 1.00 |  | 0.92- 1.08 | 9,659 | 55,720 | 1.04 | **●** | 1.02- 1.06 | |
| Respiratory Index | | | 214 | | 1,348 | | 0.95 |  | 0.82- 1.10 | 90 | 705 | 0.77 | **○** | 0.62- 0.95 | 1,091 | 5,722 | 1.14 | **●** | 1.07- 1.22 | |
| Asthma | | | 32 | | 202 | | 0.95 |  | 0.65- 1.38 | <10 | 80 | 0.45 | **○** | 0.18- 0.97 | 250 | 1,082 | 1.39 | **●** | 1.21- 1.59 | |
| Acute Bronchitis | | | 11 | | 65 | | 1.02 |  | 0.54- 1.92 | <10 | 21 | 0.57 |  | 0.09- 2.09 | 89 | 346 | 1.54 | **●** | 1.22- 1.95 | |
| COPD | | | 10 | | 97 | | 0.62 |  | 0.32- 1.19 | 18 | 150 | 0.72 |  | 0.44- 1.17 | 78 | 357 | 1.31 | **●** | 1.03- 1.67 | |
| Pneumonia | | | 19 | | 80 | | 1.43 |  | 0.86- 2.35 | 27 | 150 | 1.08 |  | 0.72- 1.63 | 36 | 158 | 1.37 |  | 0.95- 1.96 | |
| Upper Respiratory Infection | | | 49 | | 313 | | 0.94 |  | 0.70- 1.27 | <10 | 33 | 0.91 |  | 0.31- 2.20 | 531 | 3,077 | 1.04 |  | 0.94- 1.14 | |
| Respiratory Symptoms | | | 139 | | 852 | | 0.98 |  | 0.82- 1.17 | 56 | 457 | 0.74 | **○** | 0.56- 0.97 | 172 | 928 | 1.11 |  | 0.95- 1.31 | |
| Bronchitis (not specified) | | | <10 | | 33 | | 1.45 |  | 0.63- 3.05 | <10 | <10 | 0.86 |  | 0.04- 5.55 | 26 | 149 | 1.05 |  | 0.69- 1.59 | |
| Cardiovascular Index | | | 45 | | 273 | | 0.99 |  | 0.72- 1.36 | 73 | 489 | 0.90 |  | 0.70- 1.15 | 174 | 910 | 1.15 |  | 0.98- 1.35 | |
| Ischemic Heart Disease | | | <10 | | 37 | | 1.14 |  | 0.47- 2.45 | 13 | 137 | 0.57 |  | 0.32- 1.01 | 53 | 253 | 1.26 |  | 0.93- 1.69 | |
| Dysrhythmia | | | 21 | | 90 | | 1.40 |  | 0.87- 2.25 | 21 | 108 | 1.17 |  | 0.73- 1.86 | 38 | 173 | 1.32 |  | 0.93- 1.87 | |
| Congestive Heart Failure | | | <10 | | 52 | | 0.46 |  | 0.14- 1.17 | 20 | 134 | 0.90 |  | 0.56- 1.43 | 37 | 196 | 1.13 |  | 0.80- 1.61 | |
| Stroke | | | <10 | | 49 | | 1.00 |  | 0.43- 1.99 | 18 | 117 | 0.92 |  | 0.56- 1.52 | 19 | 133 | 0.86 |  | 0.53- 1.39 | |
| Diseases of Peripheral Circulation | | | 10 | | 66 | | 0.71 |  | 0.47- 1.77 | 22 | 117 | 1.13 |  | 0.72- 1.78 | 36 | 210 | 1.03 |  | 0.72- 1.46 | |
| **●** significant, positive  ***○*** significant, negative  Cells with <10 events were suppressed. When the small number could be calculated from the unexposed count and the RR, the unexposed count was also suppressed (-). | | | | | | | | | | | | | | | | | | | |  |
|  |  |  |  |  |  |  |  |  |  |  |  |  |  |  |  |  |  |  |  |  |
|  |  |  |  |  |  |  |  |  |  |  |  |  |  |  |  |  |  |  |  |  |
|  |  |  | |  | |  | | | | | | | | | | | | | |  |
|  |  |  | |  | |  | | | | | | | | | | | | | |  |
