# Supplementary material for: The San Diego 2007 wildfires and Medi-Cal emergency department presentations, inpatient hospitalizations, and outpatient visits: An observational study of smoke exposure periods and a bidirectional case-crossover analysis
Source: PLoS Med. 2018 Jul 10;15(7):e1002601. doi: 10.1371/journal.pmed.1002601 (PMC6038982; doi:10.1371/journal.pmed.1002601)
Supplement: S2 Table — RR, rate ratio. (DOCX) [file pmed.1002601.s003.docx]

| **Table S2. Age-specific rate ratios for respiratory outcomes, for very young children 0-1, young children, 2-4, young children 0-4, older children 5-17, and adults 18-64; October 22-26, 2007, San Diego County.** | | | | | | | | | | | | | | | | | | | | |
| --- | --- | --- | --- | --- | --- | --- | --- | --- | --- | --- | --- | --- | --- | --- | --- | --- | --- | --- | --- | --- |
|  | | **0-1 year** | | | **2-4 years** | | | | | **0-4 years** | | | | **5-17 years** | | | | **18-64 years** | | |
|  | | **RR** |  | **95% CI** |  | **RR** |  | **95% CI** |  | **RR** |  | **95% CI** |  | **RR** |  | **95% CI** |  | **RR** |  | **95% CI** |
| **Emergency Department Presentations** | | 1.39 | **●** | 1.15- 1.66 |  | 0.97 |  | 0.71- 1.33 |  | 1.25 | ● | 1.07- 1.47 |  | 0.79 | ○ | 0.65- 0.96 |  | 1.00 |  | 0.93- 1.08 |
| Respiratory Index |  | 1.77 | **●** | 1.32- 2.38 |  | 1.50 |  | 0.91- 2.48 |  | 1.70 | ● | 1.32- 2.19 |  | 1.19 |  | 0.80- 1.79 |  | 1.21 | ● | 1.03- 1.41 |
| Asthma |  | 3.43 | **●** | 1.49- 7.38 |  | 1.39 |  | 0.41- 3.76 |  | 2.36 | ● | 1.27- 4.39 |  | 2.00 | ● | 1.09- 3.67 |  | 1.82 | ● | 1.24- 2.67 |
| Acute Bronchitis |  | 2.95 | **●** | 1.15- 6.85 |  | 1.33 |  | 0.06- 9.03 |  | 2.56 | ● | 1.09- 5.54 |  | 1.78 |  | 0.26- 7.46 |  | 0.94 |  | 0.42- 1.88 |
| Bronchitis (not specified) |  | 0.00 |  | 0.00- 6.57 |  | 2.00 |  | 0.08- 15.92 |  | 0.89 |  | 0.04- 5.41 |  | 0.00 |  | 0.00- 8.92 |  | 1.17 |  | 0.45- 2.62 |
| Pneumonia |  | 0.84 |  | 0.13- 3.12 |  | 1.14 |  | 0.05- 7.39 |  | 0.92 |  | 0.22-2.76 |  | 1.45 |  | 0.22- 5.85 |  | 1.33 |  | 0.72- 2.46 |
| Upper Respiratory Infection |  | 1.82 | **●** | 1.25- 2.66 |  | 1.63 |  | 0.85- 3.12 |  | 1.77 | **●** | 1.28- 2.45 |  | 1.03 |  | 0.46- 2.07 |  | 0.94 |  | 0.54- 1.64 |
| Respiratory Symptoms |  | 2.06 | **●** | 1.33- 3.20 |  | 1.45 |  | 0.55- 3.31 |  | 1.91 | **●** | 1.29- 2.82 |  | 1.14 |  | 0.62- 2.09 |  | 1.22 | **●** | 1.02- 1.45 |
|  | | | | | | | | | | | | | | | | | | | | |
| **Hospital Admissions** | | 0.87 |  | 0.70- 1.09 |  | 0.82 |  | 0.44- 1.53 |  | 0.87 |  | 0.70- 1.07 |  | 0.64 | ○ | 0.46- 0.89 |  | 0.93 |  | 0.85- 1.01 |
| Respiratory Index |  | 0.99 |  | 0.46- 1.90 |  | 2.13 |  | 0.61- 6.11 |  | 1.18 |  | 0.66- 2.12 |  | 1.13 |  | 0.53- 2.18 |  | 1.11 |  | 0.92- 1.34 |
| Asthma |  | 2.18 |  | 0.49- 7.37 |  | 3.43 |  | 0.72- 13.05 |  | 2.67 |  | 0.97- 6.53 |  | 0.76 |  | 0.12- 2.79 |  | 1.44 |  | 0.87- 2.38 |
| Acute Bronchitis |  | 1.78 |  | 0.26- 7.46 |  | 0.00 |  | 0.00- 13.72 |  | 1.33 |  | 0.20- 5.28 |  | 0.00 |  | 0.00- 13.72 |  | 1.20 |  | 0.28- 3.68 |
| Bronchitis (not specified) |  | -- |  | -- |  | -- |  | -- |  | -- |  | -- |  | -- |  | -- |  | 2.29 |  | 0.33- 10.27 |
| Pneumonia |  | 1.26 |  | 0.30- 3.90 |  | 2.67 |  | 0.10- 25.02 |  | 1.45 |  | 0.43- 3.95 |  | 0.64 |  | 0.10- 2.31 |  | 1.30 |  | 0.87- 1.94 |
| Upper Respiratory Infection |  | 1.50 |  | 0.35- 4.74 |  | 0.00 |  | 0.00- 6.57 |  | 1.14 |  | 0.27- 3.49 |  | 0.00 |  | 0.00- 5.18 |  | 0.73 |  | 0.03- 4.26 |
| Respiratory Symptoms |  | 0.86 |  | 0.26- 2.24 |  | 1.33 |  | 0.06- 9.03 |  | 0.93 |  | 0.33- 2.20 |  | 1.74 |  | 0.59- 4.35 |  | 1.02 |  | 0.81- 1.29 |
|  | | | | | | | | | | | | | | | | | | | | |
| **Outpatient Presentations** | | 0.70 | **○** | 0.67- 0.74 |  | 0.63 | **○** | 0.59- 0.68 |  | 0.68 | **○** | 0.65- 0.71 |  | 0.60 | **○** | 0.57- 0.63 |  | 0.71 | **○** | 0.69- 0.73 |
| Respiratory Index |  | 1.09 |  | 0.99- 1.20 |  | 1.13 |  | 1.00- 1.28 |  | 1.11 | ● | 1.03- 1.19 |  | 0.93 |  | 0.83- 1.05 |  | 0.89 | ○ | 0.81- 0.98 |
| Asthma |  | 1.54 | **●** | 1.11- 2.12 |  | 1.18 |  | 0.91- 1.55 |  | 1.31 | ● | 1.07- 1.61 |  | 1.25 | ● | 1.05- 1.48 |  | 1.07 |  | 0.88- 1.31 |
| Acute Bronchitis |  | 1.63 | **●** | 1.21- 2.19 |  | 0.94 |  | 0.37- 2.08 |  | 1.52 | ● | 1.15- 2.00 |  | 1.25 |  | 0.64- 2.43 |  | 1.04 |  | 0.75- 1.45 |
| Bronchitis (not specified) |  | 1.17 |  | 0.45- 2.62 |  | 0.57 |  | 0.09- 2.04 |  | 0.93 |  | 0.42- 1.85 |  | 1.64 |  | 0.72- 3.39 |  | 1.20 |  | 0.76- 1.87 |
| Pneumonia |  | 1.55 |  | 0.95- 2.54 |  | 1.55 |  | 0.93- 2.56 |  | 1.55 | ● | 1.09- 2.20 |  | 0.75 |  | 0.32- 1.54 |  | 1.14 |  | 0.64- 2.04 |
| Upper Respiratory Infection |  | 1.00 |  | 0.89- 1.12 |  | 1.04 |  | 0.88- 1.21 |  | 1.01 |  | 0.92- 1.11 |  | 0.70 | ○ | 0.59- 0.84 |  | 0.75 | ○ | 0.60- 0.93 |
| Respiratory Symptoms |  | 1.34 | **●** | 1.02- 1.76 |  | 1.75 | **●** | 1.28- 2.39 |  | 1.49 | **●** | 1.22- 1.84 |  | 0.93 |  | 0.64- 1.36 |  | 0.78 | ○ | 0.64- 0.96 |

**●** significant, positive

***○*** significant, negative

-- No events occurred in the unexposed period (rate ratio undefined)
