# Supplementary material for: The San Diego 2007 wildfires and Medi-Cal emergency department presentations, inpatient hospitalizations, and outpatient visits: An observational study of smoke exposure periods and a bidirectional case-crossover analysis
Source: PLoS Med. 2018 Jul 10;15(7):e1002601. doi: 10.1371/journal.pmed.1002601 (PMC6038982; doi:10.1371/journal.pmed.1002601)
Supplement: S1 RECORD Checklist — (DOCX) [file pmed.1002601.s005.docx]

**Medi-Cal health care use during a large wildfire, San Diego 2007**

**The RECORD statement**

|  | **RECORD items** | **Location in manuscript where items are reported** |
| --- | --- | --- |
|  | RECORD 1.1: The type of data used should be specified in the title or abstract. When possible, the name of the databases used should be included.  RECORD 1.2: If applicable, the geographic region and timeframe within which the study took place should be reported in the title or abstract.  RECORD 1.3: If linkage between databases was conducted for the study, this should be clearly stated in the title or abstract. | - 1. *Provided in title and abstract.*   2. *Provided in title and abstract.*   3. *No linkages.* |
| Participants | RECORD 6.1: The methods of study population selection (such as codes or algorithms used to identify subjects) should be listed in detail. If this is not possible, an explanation should be provided.  RECORD 6.2: Any validation studies of the codes or algorithms used to select the population should be referenced. If validation was conducted for this study and not published elsewhere, detailed methods and results should be provided.  RECORD 6.3: If the study involved linkage of databases, consider use of a flow diagram or other graphical display to demonstrate the data linkage process, including the number of individuals with linked data at each stage. | *6.1. Methods sections: ‘Health data’ describes the Medi-Cal dataset; ‘Identification and description of beneficiaries’ describes selection of claims and exclusions.*  *6.2. No validation studies.*  *6.3. No linkages.* |
| Variables | RECORD 7.1: A complete list of codes and algorithms used to classify exposures, outcomes, confounders, and effect modifiers should be provided. If these cannot be reported, an explanation should be provided. | *7.1. Methods,* ‘*Identification of episodes of care and diagnoses’ provides diagnoses codes and time periods, also in Table 1.*  *‘Data analysis’ describes algorithms to classify exposures and potential effect modifiers.* |
| Data access and cleaning methods | RECORD 12.1: Authors should describe the extent to which the investigators had access to the database population used to create the study population.  RECORD 12.2: Authors should provide information on the data cleaning methods used in the study. | *12.1. Described in Methods, Health data, Identification and description of beneficiaries, and Identification of episodes of care and diagnoses.*  *12.2. Described in Methods, Health data, Identification and description of beneficiaries, and Identification of episodes of care and diagnoses.* |
| Linkage | RECORD 12.3: State whether the study included person-level, institutional-level, or other data linkage across two or more databases. The methods of linkage and methods of linkage quality evaluation should be provided. | *12.3. No linkages.* |
| Participants | RECORD 13.1: Describe in detail the selection of the persons included in the study (*i.e.,* study population selection) including filtering based on data quality, data availability and linkage. The selection of included persons can be described in the text and/or by means of the study flow diagram. | *13.1. Provided in Results, Population and Episodes of care.* |
| Limitations | RECORD 19.1: Discuss the implications of using data that were not created or collected to answer the specific research question(s). Include discussion of misclassification bias, unmeasured confounding, missing data, and changing eligibility over time, as they pertain to the study being reported. | *19.1.Misclassification (exposure) bias addressed in Discussion, prior to the summary & conclusions, lines 660-665).*  *Unmeasured confounding addressed by study design choice of case-crossover and explained in methods.*  *Use of Medi-Cal administrative dataset issues is addressed in Discussion, lines 646-568.* |
| Accessibility of protocol, raw data, and programming code | RECORD 22.1: Authors should provide information on how to access any supplemental information such as the study protocol, raw data, or programming code. | *Medi-Cal data are available from the State of California Health and Human Services Agency, Department of Health Care Services for researchers who meet the criteria for access to confidential data.*  [*http://www.dhcs.ca.gov/dataandstats/data/Pages/default.aspx*](http://www.dhcs.ca.gov/dataandstats/data/Pages/default.aspx)  *Wildfire exposure data were developed by Michigan Technological University whose*  *authors may be contacted at* [*http://www.mtri.org/*](http://www.mtri.org/)*.* |
| *Reference: Benchimol EI, Smeeth L, Guttmann A, Harron K, Moher D, Petersen I, Sørensen HT, von Elm E, Langan SM, the RECORD Working Committee. The REporting of studies Conducted using Observational Routinely-collected health Data (RECORD) Statement. *PLoS Medicine* 2015; in press.  *Checklist is protected under Creative Commons Attribution ([CC BY](http://creativecommons.org/licenses/by/4.0/)) license. | | |
